# The Missing Piece: Functional Telomerase Restored in the Beetle Model

**DOI:** 10.1093/gbe/evag069

**Published:** 2026-03-24

**Authors:** Petr Fajkus, Barbora Štefanovie, Michal Závodník, Kateřina Havlová, Miloslava Fojtová, Vratislav Peška, Jiří Fajkus

**Affiliations:** Department of Cell Biology and Radiobiology, Institute of Biophysics of the Czech Academy of Sciences, Brno 612 65, Czech Republic; Laboratory of Functional Genomics and Proteomics, National Centre for Biomolecular Research, Faculty of Science, Masaryk University, Brno 625 00, Czech Republic; Mendel Centre for Plant Genomics and Proteomics, CEITEC—Central European Institute of Technology, Masaryk University, Brno 625 00, Czech Republic; Department of Cell Biology and Radiobiology, Institute of Biophysics of the Czech Academy of Sciences, Brno 612 65, Czech Republic; Department of Cell Biology and Radiobiology, Institute of Biophysics of the Czech Academy of Sciences, Brno 612 65, Czech Republic; Laboratory of Functional Genomics and Proteomics, National Centre for Biomolecular Research, Faculty of Science, Masaryk University, Brno 625 00, Czech Republic; Mendel Centre for Plant Genomics and Proteomics, CEITEC—Central European Institute of Technology, Masaryk University, Brno 625 00, Czech Republic; Department of Cell Biology and Radiobiology, Institute of Biophysics of the Czech Academy of Sciences, Brno 612 65, Czech Republic; Laboratory of Functional Genomics and Proteomics, National Centre for Biomolecular Research, Faculty of Science, Masaryk University, Brno 625 00, Czech Republic; Mendel Centre for Plant Genomics and Proteomics, CEITEC—Central European Institute of Technology, Masaryk University, Brno 625 00, Czech Republic

**Keywords:** *Tribolium castaneum*, telomerase RNA (TR), coleoptera, scarabaeoidea, telomere maintenance and evolution, telomerase loss

## Abstract

The red flour beetle *Tribolium castaneum* yielded the first resolved telomerase reverse transcriptase structure but lacked a known telomerase RNA. We identify two telomerase RNA paralogs in *Tribolium*, confirm their expression, and reconstitute active telomerase in vitro. Extending this approach across Coleoptera, we detect telomerase RNA homologs in diverse beetle lineages, with conserved cores but lineage-specific template and telomeric DNA variants. Our findings establish *Tribolium* as a powerful model for telomerase biochemistry, illuminate the evolutionary plasticity of beetle telomere maintenance, and reveal a consistent lack of telomerase in several derived Scarabaeoidea subfamilies, pinpointing its evolutionary loss within this beetle clade.

## Graphical Abstract

**Graphical Abstract:**
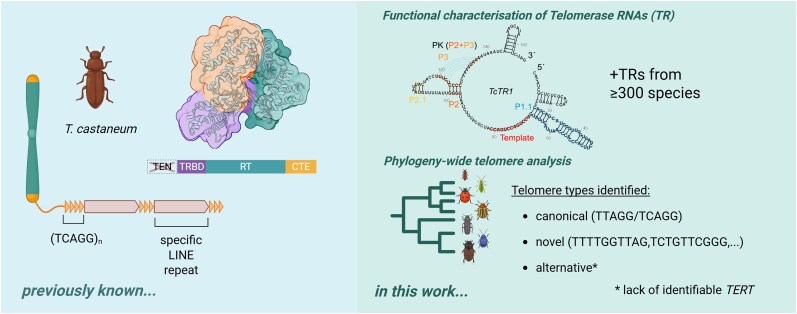

Significance Telomeres protect chromosome ends, and the enzyme telomerase replenishes them using an internal RNA template. Although beetles are the most species-rich animal order and the red flour beetle is a premier genetic model, beetle telomerase has been hard to study because its telomerase RNA was unknown. We identify two related telomerase RNA genes in *Tribolium castaneum*, confirm that they are expressed, and assemble an active telomerase enzyme in vitro. Surveying hundreds of beetle genomes, we find telomerase RNA genes across diverse beetle groups, show that changes in the RNA template match changes in telomeric DNA repeats, and pinpoint scarab beetles that likely lost telomerase.

## Introduction

Telomerase maintains chromosome ends in most eukaryotes through a ribonucleoprotein (RNP) complex composed of at least two core subunits: the telomerase reverse transcriptase (TERT) and a telomerase RNA (TR), which provides the template for telomeric repeat synthesis and serves as a scaffold for assembly of the active enzyme. While TERT is relatively conserved, TR evolves rapidly in sequence, structure, and transcriptional context, making its identification challenging across diverse lineages [reviewed in (Fajkus and Fajkus 2025)].

In recent work, we characterized TRs and mapped telomeric repeat diversity in Hymenoptera, Lepidoptera, and Trichoptera, revealing unexpected flexibility in both TR architecture and telomere composition (Fajkus et al. 2023). Hymenopteran TRs notably lack canonical features of animal TRs—such as the H/ACA box and CR4/5 domain—and are transcribed by RNA polymerase III from type 3 snRNA promoter, resembling those of plant and ciliate TRs (Hargrove et al. 1999; Fajkus et al. 2019, 2021). Lepidopteran TRs also utilize type 3 snRNA promoters (Fajkus et al. 2023), but they are transcribed by RNA polymerase II (Chou et al. 2025). These observations suggest that, while the core promoter architecture is conserved, transcriptional control and RNA structure have diversified independently across insect lineages. We also documented the plausible loss of telomerase in Cynipoidea (Hymenoptera) (Fajkus et al. 2023), which instead rely entirely on a telomerase-independent telomere maintenance mechanism—a phenomenon that has independently evolved in other groups, including Diptera, nematodes, amphibians, and yeasts (Saiga and Edstrom 1985; Biessmann et al. 1990; Yu et al. 2022; Mota et al. 2024; Brejová et al. 2025).

Here, we extend this framework to Coleoptera—the most species-rich animal order—where telomerase RNA had remained unidentified. This gap was particularly notable in *Tribolium castaneum*, a genetically tractable model species with a resolved TERT structure (Gillis et al. 2008), but no known TR. The absence of this component has limited its use in functional telomerase studies and left broader questions about telomere and telomerase evolution in beetles unresolved.

## Results and Discussion

### Identification of Telomerase RNA Genes in Coleoptera

We systematically searched for TR candidates across beetle lineages using genome screening, RNA-seq data, and comparative genomics. Candidates were required to: (i) contain a template region matching the species-specific telomeric repeat (as defined by Tandem Repeats Finder (TRFi) and telomere-to-telomere (T2T) assemblies; Tables S1 and S2); (ii) be detectably transcribed in RNA-seq libraries; (iii) have homologous sequences in related species, identified through BLASTn (Altschul et al. 1990; Camacho et al. 2009) or covariance model (CM) searches (Nawrocki and Eddy 2013).

**Fig. 1.**
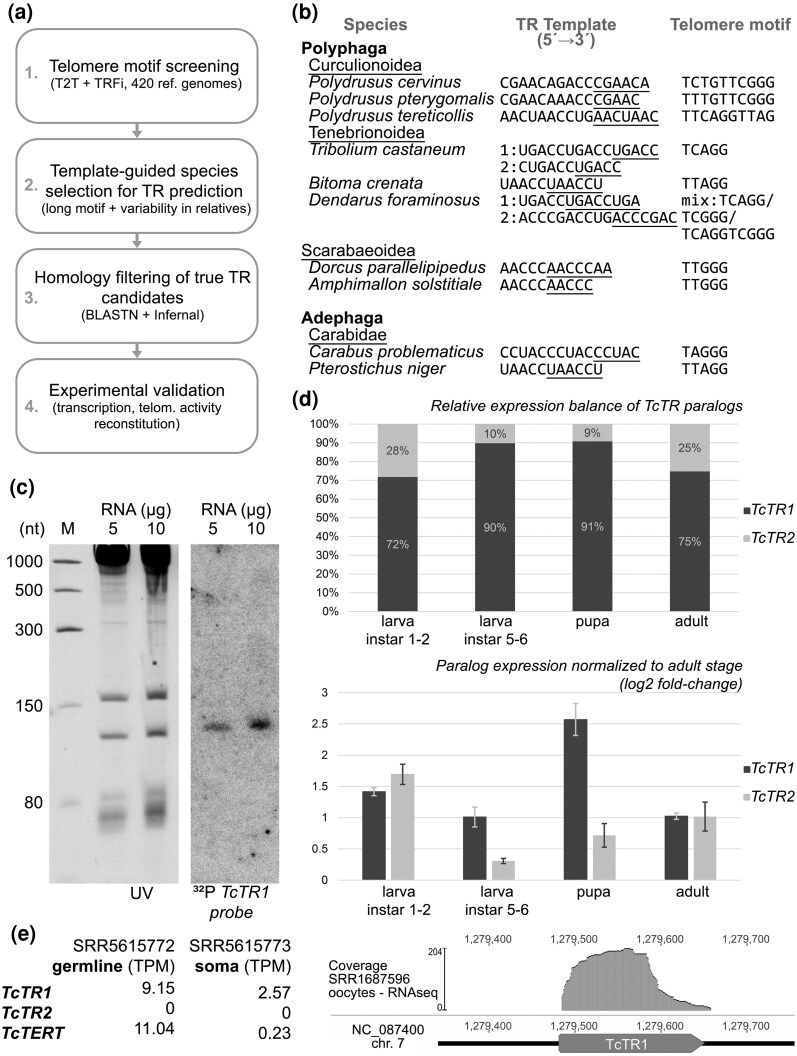
Overview of TR identification in Coleoptera and expression analyses of TR genes in *T. castaneum*. a) Summary workflow illustrating the integrated approach used for TR discovery, including telomere repeat inference (TRFi, T2T assemblies), template-guided searches in selected genomes, BLAST- and Infernal-based homology screening, and experimental validation in the *T. castaneum* model. b) Comparison of TR template regions with the corresponding telomeric repeat motifs across representative beetle lineages with variable telomeres, demonstrating strong template-telomere correspondence. Annealing template portion is underlined. c) Northern hybridization using total RNA from adult beetles hybridized with radiolabelled TcTR1 probe. Gels were stained with SYBR™ Gold (S11494, Thermo) and visualized in UV light (UV). Low range ssRNA Ladder (N0364S, New England Biolabs) was used as a marker (M). d) Relative expression balance of TcTR1 and TcTR2 across developmental stages (upper panel) and expression normalized to the adult stage using β-actin gene as reference gene (Sang et al. 2015) (lower panel), demonstrating differential paralog usage during development. e) TcTR and TcTERT transcript levels in germline and somatic tissues (TPM values for SRR5615772 and SRR5615773 datasets) and RNA-seq read coverage across the TcTR1 locus (using SRR1687596 dataset), supporting TR transcription and transcript boundary prediction.

To initiate cross-species searches, genomes of related species with variable telomere repeats (inferred from TRFi/T2T, Tables S1 and S2) were screened for template-like sequences with 200 nt of flanking context (Fig. 1a). In *Polydrusus* (Curculionidae), three genomes, each with a distinct telomere sequence, yielded high-confidence TR candidates (Fig. 1b). These were used in iterative BLASTn searches to recover orthologs from progressively more distant taxa. High-confidence sequences from *Polydrusus* and other related genera from Curculionidae were aligned to construct CMs in Infernal (Nawrocki and Eddy 2013), which were iteratively refined with newly recovered high-scoring hits and applied across Coleoptera genome assemblies, enabling detection of TR homologs even in highly divergent lineages. In *T. castaneum*, transcript boundaries for identified TR homologs were subsequently mapped using RNA-seq datasets (Fig. 1e). In total, we identified TR candidates in 301 species from 420 analysed, representing all major coleopteran families (summarized in Table S2).

In *Tribolium castaneum*, we identified two telomerase RNA (TR) paralogs, TcTR1 and TcTR2 (76% sequence identity). Both are transcribed, as supported by rRNA-depleted RNA-seq from TERT-positive samples, and validated by RT-qPCR and northern blot (Fig. 1c and d), with developmental stage- and tissue-specific expression levels.

**Fig. 2.**
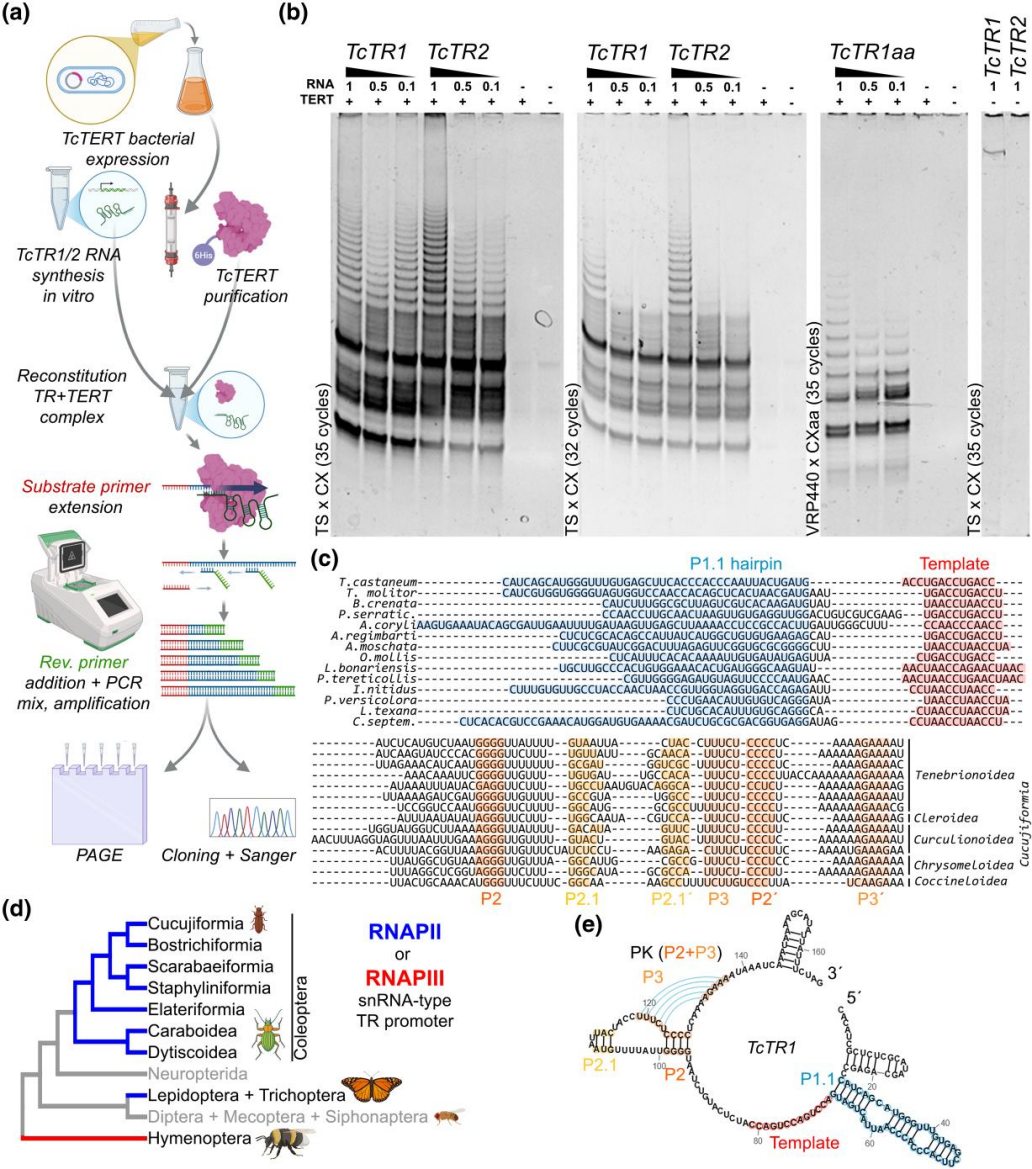
Functional reconstitution of *T. castaneum* telomerase and conserved structural features of beetle TRs. a) Overview of the experimental workflow for *T. castaneum* telomerase reconstitution, including bacterial expression and purification of TcTERT, in vitro synthesis of TcTR paralogs, assembly of the TR-TERT complex, substrate-primer extension, and TRAP amplification followed by PAGE and Sanger sequencing of TRAP products. b) TRAP assays demonstrating telomerase activity reconstituted with TcTERT and either TcTR1, TcTR2, or the template-mutant TcTR1aa. Characteristic amplification ladders are observed for both paralogs and the mutant, with activity decreasing in RNA dilution series (1, 0.5, 0.1)—more apparent upon reduction of PCR cycles (32 cycles). No amplification is detected in negative controls lacking TERT or TR RNA or both. c) Alignment of representative beetle TR sequences from the infraorder Cucujiformia, highlighting conserved structural elements, including the P1.1 hairpin (blue), the P2 region and P3 regions forming the core pseudoknot, the inner pseudoknot element P2.1 (yellow), and the template region (red). Taxonomic affiliations of each sequence are shown on the right. d) Phylogenetic distribution of snRNA-type promoters associated with TR loci in insect species for which TRs have been identified. e) Predicted secondary structure of TcTR1 [using RNAcanvas (Johnson and Simon 2023)], illustrating the conserved core domains, including the P1.1 stem, the pseudoknot region (P2/P3), and the template region. Colors correspond to the alignment in panel (c).

Promoter inspection revealed upstream architecture characteristic of type-3 snRNA genes transcribed by RNA polymerase II (Fig. 2d, Fig. S1a**)** consistent with RNAPII-mediated TR transcription in Lepidoptera. Corresponding promoter signatures were observed for additional beetle TRs (Fig. S1). Compared to Lepidopteran TRs, Coleoptera exhibit extreme sequence divergence, making alignments across distant species difficult. Nevertheless, the core structural elements exemplified by *TcTR1* (Fig. 2c and e)—including the Template Boundary Element (TBE/P1.1), the pseudoknot (PK), and the inter-pseudoknot stem-loop P2.1—are conserved in beetles, paralleling those in Lepidoptera.

### Functional Reconstitution of *Tribolium* Telomerase

Recombinant *T. castaneum* TERT combined with in vitro-transcribed TcTR1 or TcTR2 produced clear TRAP ladders, with no activity in negative controls (Fig. 2b). The TcTR dilution series (1x, 2x, 10x) exhibited proportional signal loss, more pronounced at 32 PCR cycles, reflecting decreased telomerase activity and fewer telomerase-extended substrates. The template-mutant TcTR1aa (carrying G→a substitutions; 5´-TaACCTaACCTaACC-3´) also supported detectable activity. Cloned and Sanger-sequenced TRAP products (Fig. S2) matched the expected telomeric repeat sequences, ruling out primer-derived artefacts.

### Evolutionary Diversity of Telomeric Repeats and Maintenance Mechanisms in Coleoptera

Beyond previously reported telomeric motifs such as TTAGG, TCAGG, and TTTGGG (Prusakova et al. 2021), our reanalysis reveals much broader diversity of telomeric sequences across Coleoptera. Using TRFi on raw genomic data, together with T2T assemblies and long-read datasets, we identified numerous lineage-specific telomeric variants. Importantly, shifts in telomere sequence were consistently mirrored by corresponding changes in the TR template region, reinforcing the functional link between TR evolution and telomere composition (examples in Fig. 1b; full list in Table S2).

In several coleopteran lineages, telomeric motifs, TR candidates, and TERT homologs could not be detected despite relaxed tblastn searches using multiple phylogenetically diverse TERT queries (Fig. S3a; Supplementary Methods S8; Table S2). While incomplete assemblies or extreme sequence divergence cannot be excluded, the concurrent absence of telomeric repeats, TR, and TERT may indicate lineage-specific telomerase loss. Similar losses have been reported in Diptera (Biessmann et al. 1990; Lopez et al. 1996), *Meloidogyne* spp. (Nematoda) (Mota et al. 2024), *Pleurodeles waltl* (Vertebrata) (Yu et al. 2022), and cynipoid wasps (Hymenoptera) (Fajkus et al. 2023).

Notably, genome-wide TERT screening frequently “failed” in Scarabaeoidea despite the high number of available genome assemblies (Fig. S3a), motivating a focused phylogeny-assisted analysis of this group. Within Scarabaeoidea, TERT showed a clear phylogenetic pattern (Fig. S3b): while TERT-like sequences were recovered in basal Melolonthinae, no significant TERT hits were obtained in the derived lineages Dynastinae, Rutelinae, Cetoniinae, and Hopliini, despite extensive screening of genome assemblies, transcriptomic data, and raw sequencing reads. This systematic absence is consistent with a phylogenetic stratum of TERT loss, although extreme sequence divergence cannot be fully excluded.

In *Cetonia aurata* (Cetoniinae), where both T2T and PacBio data are available, analyses of chromosome termini and TeloSearchLR (Chung et al. 2025) independently revealed enrichment of ∼64-nt satellite repeat, that may represent telomere DNA (Fig. S4), supporting a non-telomerase–based mode of telomere maintenance.

## Conclusions

We identified beetle telomerase RNAs and reconstituted active telomerase in *T. castaneum*, demonstrating that beetle TERTs remain catalytically competent in vitro despite lacking the canonical N-terminal (TEN) domain found in most other eukaryotes. Structure–function studies in yeast, ciliates, and vertebrates show that the TEN domain facilitates RNA and DNA binding, enhances processivity, and recruits accessory subunits (Friedman et al. 2003; Eckert and Collins 2012; Akiyama et al. 2015). How this minimalist enzyme assembles and functions in vivo remains unresolved, and beetles may rely on additional cofactors to stabilize or regulate the complex—candidates that can now be explored using the TR knowledge presented here. Importantly, the precise correspondence between TR templates and telomeric repeat variants—most evident in Curculionidea—demonstrates that evolutionary changes in TRs have directly shaped telomere motif diversity, establishing telomerase as a central force in telomere evolution and maintenance in beetles.

## Materials and Methods

### Beetle Culture and RNA Extraction

*Tribolium castaneum* (strain San Bernardino) was reared at 32 °C in constant darkness on flour diet as described in the Beetle Book (https://wwwuser.gwdg.de/∼gbucher1/beetle-book1.pdf). Developmental stages were collected by sifting (300 µm for early larvae, 800 µm for late larvae, pupa, and adult stages. Total RNA from larvae (L1, L4), pupae, and adults was extracted using TRI Reagent (TR 118, Molecular Research Center) and assessed on a TapeStation 4150 (Agilent). RNA samples were used for TcTR1/TcTR2 expression analyses by RT-qPCR and northern blot (Supplementary Methods S1 and S2, Fig. S5).

### Systematic Search for Telomeric Repeats and TERT

Telomeric repeat motifs were predicted using TRFi on selected Illumina WGS datasets and independently on 2,000-nt terminal regions of available T2T assemblies at NCBI, following procedures established in Hymenoptera (Fajkus et al. 2023). TeloSearchLR analysis of *Cetonia aurata* raw PacBio data is described in Supplementary Methods S7. TERT detection was performed across genome assemblies using an approach analogous to (Fajkus et al. 2023) and extended to Scarabaeoidea-derived TSA and SRA datasets (Dietz et al. 2023). Species were interpreted in their phylogenetic context (Dietz et al. 2023), allowing tracking of shared presence or absence of TERT among closely related scarabaeoid lineages. Detailed parameters are provided in Supplementary Methods S8, and results are summarized in Fig. S3.

### TR Prediction and Comparative Genomics

Building on telomere repeat data obtained from TRFi and T2T analyses (Tables S1 and S2), genomes of three *Polydrusus* species (Curculionidae) with distinct telomere sequences were searched in Geneious R8 (https://www.geneious.com) for template-like regions together with 200 nt of flanking context. BLASTn comparisons (word size = 11; max E-value = 1e−3; gap penalties = 5/2) recovered several candidates, but only a single sequence was consistently identified in homolog searches across related telomere-variable Curculionoidea, designating it as the most plausible TR candidate. These high-confidence homologs were aligned to build and iteratively refine covariance models in Infernal (Nawrocki and Eddy 2013), which were subsequently applied across Coleoptera genome assemblies (optimized CM available at Zenodo, doi:10.5281/zenodo.17782128). Transcript boundaries for *T. castaneum* TRs were inferred from predicted promoter positions (Fig. S2, Supplementay Methods S6) and supported by mapped RNA-seq dataset from oocytes (SRR1687596) (Ninova et al. 2016) as described in Supplementary Methods S3.

### Functional Validation

TR expression in *T. castaneum* was assessed by RT-qPCR at four developmental stages (Supplementary Methods S2), northern blot (Supplementary Methods S1), and analysis of RNA-seq datasets from the study (Lewis et al. 2018) comprising somatic-specific (SRR5615772) and germline-specific (SRR5615773) female samples. Mapping and quantification of TcTR1, TcTR2, and TERT expression levels were performed using STAR and RSEM, with full details in Supplementary Methods S3. Telomerase reconstitution assays were adapted from a previously optimized protocol for in vitro telomerase expression and activity detection (Schuller et al. 2011), with the major modification that TERT was reconstituted not with total RNA but with in vitro–transcribed TcTR1 or TcTR2. Protocol specifics, construct designs, purification steps, PCR conditions and template mutagenesis (in case of *TcTR1aa*) are provided in Supplementary Methods S4, S5, and S9.

## Supplementary Material

evag069_Supplementary_Data

## Data Availability

All data supporting the findings of this study are included in the Supplementary Information and are publicly available via the Zenodo repository (doi:10.5281/zenodo.17782128).

## References

[evag069-B1] Akiyama BM, Parks JW, Stone MD. The telomerase essential N-terminal domain promotes DNA synthesis by stabilizing short RNA-DNA hybrids. Nucleic Acids Res. 2015:43:5537–5549. 10.1093/nar/gkv406. 25940626 PMC4477650

[evag069-B2] Altschul SF, Gish W, Miller W, Myers EW, Lipman DJ. Basic local alignment search tool. J Mol Biol. 1990:215:403–410. 10.1016/S0022-2836(05)80360-2. 2231712

[evag069-B3] Biessmann H, et al Addition of telomere-associated het DNA-sequences heals broken chromosome ends in drosophila. Cell. 1990:61:663–673. 10.1016/0092-8674(90)90478-W. 2111731

[evag069-B4] Brejová B, et al 2025 Sep 12. Noncanonical chromosomal-end-specific telomeric arrays in naturally telomerase-negative yeasts [preprint]. bioRxiv:2025.2009.2007.674783. 10.1101/2025.09.07.674783 PMC13234504 42240619

[evag069-B5] Camacho C, et al BLAST+: architecture and applications. BMC Bioinformatics. 2009:10:421. 10.1186/1471-2105-10-421. 20003500 PMC2803857

[evag069-B6] Chou YS, et al A degenerate telomerase RNA directs telomeric DNA synthesis in lepidopteran insects. Proc Natl Acad Sci U S A. 2025:122:e2424443122. 10.1073/pnas.2424443122. 40020192 PMC11892584

[evag069-B7] Chung G, Piano F, Gunsalus KC. TeloSearchLR: an algorithm to detect novel telomere repeat motifs using long sequencing reads. G3 (Bethesda). 2025:15:jkaf062. 10.1093/g3journal/jkaf062. 40169380 PMC12134996

[evag069-B8] Dietz L, et al A transcriptome-based phylogeny of Scarabaeoidea confirms the sister group relationship of dung beetles and phytophagous pleurostict scarabs (Coleoptera). Syst Entomol. 2023:48:672–686. 10.1111/syen.12602.

[evag069-B9] Eckert B, Collins K. Roles of telomerase reverse transcriptase N-terminal domain in assembly and activity of Tetrahymena telomerase holoenzyme. J Biol Chem. 2012:287:12805–12814. 10.1074/jbc.M112.339853. 22367200 PMC3339965

[evag069-B10] Fajkus P, et al Telomerase RNAs in land plants. Nucleic Acids Res. 2019:47:9842–9856. 10.1093/nar/gkz695. 31392988 PMC6765143

[evag069-B11] Fajkus P, et al Evolution of plant telomerase RNAs: farther to the past, deeper to the roots. Nucleic Acids Res. 2021:49:7680–7694. 10.1093/nar/gkab545. 34181710 PMC8287931

[evag069-B12] Fajkus P, et al Telomerase RNA in Hymenoptera (Insecta) switched to plant/ciliate-like biogenesis. Nucleic Acids Res. 2023:51:420–433. 10.1093/nar/gkac1202. 36546771 PMC9841428

[evag069-B13] Fajkus P, Fajkus J. Telomerase RNA evolution: a journey from plant telomeres to broader eukaryotic diversity. Biochem J. 2025:482:169–179. 10.1042/BCJ20240501. 39889303 PMC12133305

[evag069-B14] Friedman KL, Heit JJ, Long DM, Cech TR. N-terminal domain of yeast telomerase reverse transcriptase: recruitment of Est3p to the telomerase complex. Mol Biol Cell. 2003:14:1–13. 10.1091/mbc.e02-06-0327. 12529422 PMC140223

[evag069-B15] Gillis AJ, Schuller AP, Skordalakes E. Structure of the *Tribolium castaneum* telomerase catalytic subunit TERT. Nature. 2008:455:633–637. 10.1038/nature07283. 18758444

[evag069-B16] Hargrove BW, et al Identification of an essential proximal sequence element in the promoter of the telomerase RNA gene of Tetrahymena thermophila. Nucleic Acids Res. 1999:27:4269–4275. 10.1093/nar/27.21.4269. 10518620 PMC148703

[evag069-B17] Johnson PZ, Simon AE. RNAcanvas: interactive drawing and exploration of nucleic acid structures. Nucleic Acids Res. 2023:51:W501–W508. 10.1093/nar/gkad302. 37094080 PMC10320051

[evag069-B18] Lewis SH, et al Pan-arthropod analysis reveals somatic piRNAs as an ancestral defence against transposable elements. Nat Ecol Evol. 2018:2:174–181. 10.1038/s41559-017-0403-4. 29203920 PMC5732027

[evag069-B19] Lopez CC, Nielsen L, Edstrom JE. Terminal long tandem repeats in chromosomes from Chironomus pallidivittatus. Mol Cell Biol. 1996:16:3285–3290. 10.1128/MCB.16.7.3285. 8668143 PMC231322

[evag069-B20] Mota APZ, et al Unzipped genome assemblies of polyploid root-knot nematodes reveal unusual and clade-specific telomeric repeats. Nat Commun. 2024:15:773. 10.1038/s41467-024-44914-y. 38316773 PMC10844300

[evag069-B21] Nawrocki EP, Eddy SR. Infernal 1.1: 100-fold faster RNA homology searches. Bioinformatics. 2013:29:2933–2935. 10.1093/bioinformatics/btt509. 24008419 PMC3810854

[evag069-B22] Ninova M, Ronshaugen M, Griffiths-Jones S. MicroRNA evolution, expression, and function during short germband development in *Tribolium castaneum*. Genome Res. 2016:26:85–96. 10.1101/gr.193367.115. 26518483 PMC4691753

[evag069-B23] Prusakova D, et al Telomeric DNA sequences in beetle taxa vary with species richness. Sci Rep. 2021:11:13319. 10.1038/s41598-021-92705-y. 34172809 PMC8233369

[evag069-B24] Saiga H, Edstrom JE. Long tandem arrays of complex repeat units in chironomus telomeres. EMBO J. 1985:4:799–804. 10.1002/j.1460-2075.1985.tb03700.x. 4006906 PMC554259

[evag069-B25] Sang W, He L, Wang XP, Zhu-Salzman K, Lei CL. Evaluation of reference genes for RT-qPCR in *Tribolium castaneum* (Coleoptera: Tenebrionidae) under UVB stress. Environ Entomol. 2015:44:418–425. 10.1093/ee/nvv010. 26313197

[evag069-B26] Schuller AP, Harkisheimer MJ, Skordalakes E. In vitro reconstitution of the active T. castaneum telomerase. J Vis Exp. 2011:53:e2799. 10.3791/2799. PMC3196162 21775967

[evag069-B27] Yu Q, et al 2022 Mar 26. Telomerase-independent maintenance of telomere length in a vertebrate [preprint]. bioRxiv:2022.2003.2025.485759. 10.1101/2022.03.25.485759

