# Correction to: The Missing Piece: Functional Telomerase Restored in the Beetle Model

**DOI:** 10.1093/gbe/evag100

**Published:** 2026-04-20

This is a correction to: Petr Fajkus, Barbora Štefanovie, Michal Závodník, Kateřina Havlová, Miloslava Fojtová, Vratislav Peška, Jiří Fajkus, The Missing Piece: Functional Telomerase Restored in the Beetle Model, *Genome Biology and Evolution*, Volume 18, Issue 3, March 2026, evag069, https://doi.org/10.1093/gbe/evag069

Under subheading **Evolutionary Diversity of Telomeric Repeats and Maintenance Mechanisms in Coleoptera**, Supplementary Figure S3 was referenced incorrectly in the first sentence as: “In several coleopteran lineages, telomeric motifs, TR candidates, and TERT homologs could not be detected despite relaxed tblastn searches using multiple phylogenetically diverse TERT queries (Fig. S1a[…]”. This is emended to read “Fig. S3a”.

Beneath the same subtitle, in the third paragraph, in text reading: “Notably, genome-wide TERT screening frequently “failed” in Scarabaeoidea despite the high number of available genome assemblies (Fig. S1a), motivating a focused phylogeny-assisted analysis of this group. Within Scarabaeoidea, TERT showed a clear phylogenetic pattern (Fig. S1b):[…]” figure references are corrected to read instead: “Fig.S3a” and “Fig. S3b” respectively.

In section **Material and Methods,** in the last sentence of the paragraph under subheading **Systematic Search for Telomeric Repeats and TERT**: “Detailed parameters are provided in Supplementary Methods S8, and results are summarized in Fig. S1.” the reference is corrected to read: “Fig. S3”.

The emendations have been made within the paper.

