# Supplementary material for: The Missing Piece: Functional Telomerase Restored in the Beetle Model
Source: Genome Biol Evol. 2026 Mar 24;18(3):evag069. doi: 10.1093/gbe/evag069 (PMC13023368; doi:10.1093/gbe/evag069)
Supplement: evag069_Supplementary_Data [file evag069_supplementary_data.zip › Supplementary_Methods_and_Figures.pdf]

The Missing Piece: Functional Telomerase Restored in the Beetle Model

Supplementary Methods

S1 Northern blot

Northern blot analysis was performed essentially as described in [1]. Briefly, 5-10 µg of total RNA were mixed with RNA loading dye (B0363S, New England Biolabs), denatured for 5 min at 65 °C, and separated on 7% denaturing polyacrylamide gels containing 8 M urea in 1x TBE. Electrophoresis was run initially at 100 V and subsequently at 150 V in a Mini-PROTEAN system (Bio-Rad) with constant heating (45 °C). After staining with SYBR™ Gold (S11494, Thermo), RNAs were transferred onto Hybond-XL membranes (GE Healthcare) using the Trans-Blot® Turbo™ transfer system (Bio-Rad). Membranes were rinsed in 1x TBE and hybridized overnight at 55 °C with the [^{32}P]-labelled dsDNA TcTR1 probe, prepared using the DecaLabel DNA Labelling Kit (FERK0622,Thermo). Signals were visualized using a phosphorimager FLA-7000 (GE Healthcare). Specific primers used for generating the TcTR1 probe template are listed in **S9 (Primers and Constructs)**.

S2 TcTR1 and TcTR2 RT-qPCR

Tribolium samples were collected at four developmental stages: young larvae (instars I-II; ~30-40 individuals per biological replicate), old larvae (instars V-VI; ~15 individuals per replicate), pupae (10 individuals per replicate), and adults (10 individuals per replicate), with three biological replicates analyzed per stage. Total RNA was isolated using the TRI Reagent (TR 118, Molecular Research Center). Samples were lysed in 0.75 ml TRI Reagent buffer, phase separation was performed with 0.1 ml BCP (1-bromo-3-chloropropane), and RNA was precipitated with 0.5 ml isopropanol, washed with 75% ethanol, air-dried, and dissolved in DEPC-treated water (up to 100 µl). Residual genomic DNA was removed using the TURBO DNA-free™ kit (AM1907, Thermo), and the final RNA volume was adjusted to 40 µl. RNA concentration was measured using a Qubit fluorometer (Thermo).

For cDNA synthesis, 1 µg of total RNA was reverse-transcribed using M-MuLV reverse transcriptase (28025021, Thermo) and random nonamers (R7647, Sigma). The resulting cDNA was diluted 2x prior to quantitative PCR.

RT-qPCR was performed for TcTR1 and TcTR2 using β-actin as the reference gene for normalization. Primer sequences are listed in part **S9**. Reactions were prepared in a total volume of 20 µl containing 10 µl Luna Universal qPCR Master Mix (M3003L, New England Biolabs), 0.5 µl 10 µM forward primer, 0.5 µl 10 µM reverse primer, 1 µl of 2x diluted cDNA, and 8 µl nuclease-free water. Amplification was carried out on a Rotor-Gene 6000 (Qiagen) instrument using the manufacturer's recommended protocol: initial denaturation at 95 °C for 60 s; followed by 45 cycles of 95 °C for 15 s and 60 °C for 30 s; and a melt-curve analysis from 60 to 95 °C. Reaction performance and amplification efficiencies are shown in **Supplementary Material S5**. Relative expression levels were calculated using the ΔΔCt method using technical triplicates for each biological replicate.

S3 RNA-seq analyses

SRA files SRR5615772 and SRR5615773 containing paired-end raw Illumina transcriptomic reads, reference genome Tribolium_castaneum.Tcas5.2.dna.toplevel.fa.gz and reference genome annotation Tribolium_castaneum.Tcas5.2.61.gtf were downloaded from www.ebi.ac.uk. Adapters removal and pre-processing of raw reads was done using Trimmomatic v038 [2] with the following settings: SLIDINGWINDOW:4:15, HEADCROP:12, MINLEN:35. The data was cleaned from ribosomal RNA reads by sortmerna v4.3.7 using RNA database smr_v4.3_default_db [3]. The annotation Tribolium_castaneum.Tcas5.2.61.gtf was modified by adding TR_ncRNA1 and TR_ncRNA2 coordinates and sorted. Next, pre-processed reads were mapped to the reference genome and transcriptome using STAR v2.7.10b [4]. Gene counts were quantified with RSEM v1.3.1. with the settings: --paired-end, --strandedness reverse, --seed 123456, --estimate-rspd, --calc-ci [5].

S4 Reconstitution of telomerase activity, TRAP assay
To generate the pET28a-6xHis-TEV-3xFLAG-TcTERT expression construct, TcTERT cDNA was PCR-amplified from pEX-A258-3xFLAG-TcTERT (ordered from Eurofins; see part **S9**) using primers prBS191 and prBS192 (**S9**) and cloned into the NdeI site of the pET28a vector using NEBuilder HiFi DNA Assembly (E2621, New England Biolabs).
The 6xHis-TEV-3xFLAG-TcTERT protein was expressed in *E. coli* Rosetta(DE3)pLysS cells (70956-M,Sigma and purified as described in [6], with the following modifications. Affinity purification was performed on a HisTrap FF column (Cytiva) using a linear imidazole gradient (15–300 mM), followed by size-exclusion chromatography on a Superdex 200 column (Cytiva). Peak fractions were concentrated using Amicon Ultra 30 kDa centrifugal filters (UFC5030, Sigma), aliquoted, flash-frozen in liquid nitrogen, and stored at -80 °C until use.
Purification was monitored by SDS-PAGE (**Picture 1**), showing separation of the Superdex 200 A9 fraction, with BSA included as a concentration control. Protein identity was further verified by western blotting (**Picture 2**). Proteins were transferred to a nitrocellulose membrane for 1.5 h at 350 mA. The membrane was blocked in 5% milk in TBST for 1 h at room temperature and incubated with monoclonal anti-polyhistidine antibody (mouse; 1:3000; H1029, Sigma H1029) for 1 h at room temperature. After three washes in TBST, the membrane was incubated with HRP-conjugated anti-mouse IgG (goat; 1:5000; A0168, Sigma) for 1 h at room temperature and washed three times in TBST. Signal detection was performed using SuperSignal™ West Dura Extended Duration Substrate (34075, Thermo) with a 45 s exposure.

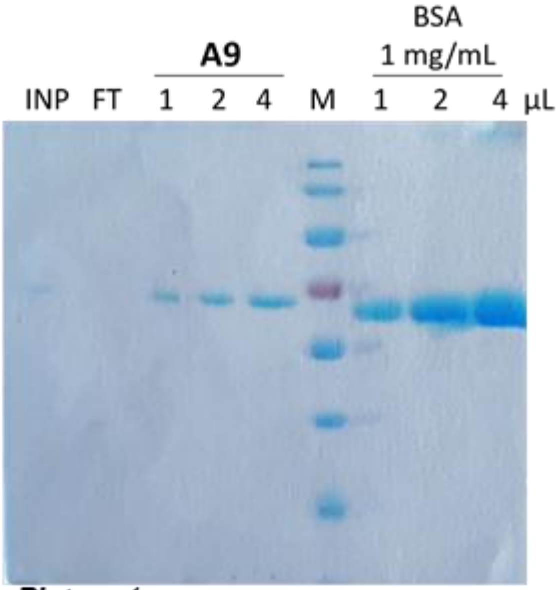

Picture 1

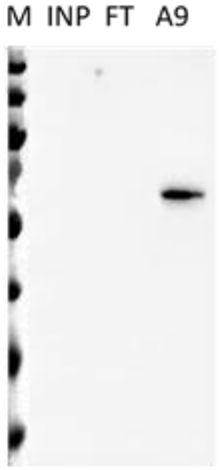

Picture 2

RNA synthesis was performed using the HiScribe® T7 Quick High Yield RNA Synthesis Kit (E2050S, New England Biolabs). Approximately 150 ng of purified PCR products (cleaned using SPRIselect beads (B23318, Beckman Coulter) corresponding to T7-TcTR1, T7-TcTR2, or T7-TcTR1aa were used as templates. Reactions were carried out in 30 µl volumes according to the manufacturer's protocol, followed by DNase I treatment using the kit-provided enzyme and RNA cleanup with SPRIselect beads. RNAs were disolved in 1xTE buffer.

For telomerase assembly, approx 4 µg of purified TERT protein and RNA dilutions (1x, 5x, 10x), corresponding to 140, 28, or 14 ng of TcTR1, TcTR2, or TcTR1aa, were mixed in assembly buffer containing 25 mM Tris-HCl (pH 8.0), 200 mM KCl, 10% glycerol, 5 mM β-mercaptoethanol, and 10 mM imidazole (pH 7.5). Reaction mixtures were incubated for 2 h at 22 °C.

The reconstituted telomerase was then combined with 1 µM Tcas_TS primer (or VRP440 for TcTR1aa; **S9**) in elongation buffer containing 20 mM Tris–HCl (pH 8.0), 7.5 mM $MgCl_2$, 63 mM KCl, 0.05% Tween 20, 1 mM EGTA, 0.01% BSA, and 0.5 mM dNTPs. Telomerase-mediated

primer elongation was carried out for 1 h at 30 °C with gentle shaking (350 rpm). All oligonucleotides and constructs are included in **Supplementary Methods S9**.
Elongation products were PCR-amplified using Q5 High-Fidelity DNA Polymerase (M0491L , New England Biolabs) with the Tcas_TS and Tcas_CX primers (or VRP440 and Tcas_CXaa primers for TcTR1aa; **S9**). PCR conditions were as follows: initial denaturation at 95 °C for 2 min; 35 cycles (or 32 cycles) of 95 °C for 10 s, 65 °C for 30 s, and 72 °C for 30 s; and a final extension at 72 °C for 5 min. PCR products were resolved on a 12.5% polyacrylamide gel in 0.5x TBE buffer and stained with GelStar Nucleic Acid Gel Stain (50535, Lonza).
To characterize telomerase extension products, TRAP products were A-tailed using MyTaq DNA Polymerase (BIO-21106, Bioline), cloned using a TOPO TA Cloning Kit (K457501, Invitrogen), and Sanger-sequenced (Eurofins).

S5 Template mutagenesis
Using pEX-A258–TcTR1 as a template, site-directed mutagenesis was performed to generate the TcTR1aa variant with primers TcTR1_aaF and TcTR1_aaR (**S9**) using the QuikChange Site-Directed Mutagenesis Kit (200518, Agilent). Mutant transformants were confirmed by Sanger sequencing (Eurofins) and used for in vitro transcription and telomerase reconstitution assays.

S6 snRNA promoter analysis
Type 3 snRNA promoters were characterized as described in [7]. Covariance models (CMs) for U1 (RFAM: RF00003), U2 (RF00004), U3 (RF00012), U4 (RF00015), U5 (RF00020), U6 (RF00026), U6atac (RF00619), U8 (RF00096), U11 (RF00548), MRP (RF00030), and 7SK (RF01052) were used to identify type 3 promoter housekeeping RNAs in individual genome assemblies. Significant hits were extended with 200 nt of genomic context (full list available at Zenodo, doi:10.5281/zenodo.17782128). In representative species, promoter regions of these RNAs were aligned with the promoter region of the telomerase RNA (TR) in Geneious R8 (https://www.geneious.com) to assess whether the TR promoter is likely transcribed by RNA polymerase II or RNA polymerase III.

S7 TeloSearchLR analysis
TeloSearchLR was installed on the MetaCentrum national grid computing infrastructure as a Conda environment (telosearchlr-env) following the developer’s recommendations (https://github.com/gchchung/TeloSearchLR). A publicly available PacBio HiFi whole-genome sequencing dataset of *Cetonia aurata* (SRA: ERR10753927) was analyzed. To detect tandem repeats of different unit lengths, three independent TeloSearchLR runs were performed targeting repeat sizes of 5–20 bp, 21–50 bp, and 51–100 bp. Output plots were inspected manually, and profiles showing a telomere-like signature-defined as a strong enrichment of repeats at the beginning or end of long reads-were selected. These candidate terminal repeats were subsequently compared with chromosome-end repeat enrichment in the T2T genome assembly (GCA_949128085.1).

S8 TERT search across Coleoptera
TERT detection across Coleoptera genomes was performed using a tblastn-based approach analogous to that described for Hymenoptera in [7]. TERT protein queries (listed in **Supplementary Material S1**) were used for independent tblastn searches against representative Coleoptera genome assemblies at NCBI (word size = 3; gap costs = 11/1; E-value threshold = 1e-4). The results were processed using python and bash scripting and visualized as E-value heatmaps, corresponding codes and raw datasets are available as a zip archive at Zenodo (doi:10.5281/zenodo.17782128). TERT detection within Scarabaeoidea was further examined using available transcriptomic (TSA) data, as indicated next to the phylogenetic tree (**Supplementary Material S1B**), and three Scarabaeoidea-derived TERT queries (queries 3, 11, and 12). In addition, raw PacBio datasets from cetoniine genomes with available T2T assemblies (Cetonia aurata, Protaetia

brevitarsis) were screened. To increase sensitivity, translated TERT CDS fragments from closely related Melolonthidae species (accession numbers listed in **Supplementary Material S1B**) were also used as queries in blast searches.

S9 Primers and constructs

primer name	5´ - 3´	notes
TcTR1_rev	CTAGAAATAATATGCTTTTAT	PCR template for RNA synthesis
T7_TcTR1_start	TAATACGACTCACTATAGGGCACATCGCTCTCGCA	
TcTR2_rev	AAGTGGAATATGCTTTGATT	PCR template for RNA synthesis
T7_TcTR2_start	TAATACGACTCACTATAGGGCATCGCTCTCACAGA	
TcTR1_aaR	CATTAGACATGAGATGGTTAGGTTAGGTTATCAGTAATTGGGTGGGTGAAGC	Template mutagenesis
TcTR1_aaF	GCTTCACCCACCCAATTACTGATAACCTAACCTAACCATCTCATGTCTAATG	
TcACTb_fw	TCCATCATGAAGTGCGATGT	qPCR actin
TcACTb_rev	CCACATCTGTTGGAATGTCG	
qTcTR1_F4	AGCATGGGTTTGTGAGCTT	qPCR TcTR1
qTcTR1_R1	TCTTTTGAGGGGAGAAAGGT	
qTcTR2_F2	GTTCGTTCAATGTGCAGTTC	qPCR TcTR2
qTcTR2_R3	GAGGGGAGAAATGTAGCAATTAC	
prBS191	GCCGCGCGGCAGCCATGACTACAAAGACCATGACG	TcTERT clonning
prBS192	CCACCAGTCATGCTAGCCATTAAATTGAGGCCTTTCTG	
Tcas_TS	AAGCCGTCGAGCAGAGTC	TRAP (TcTR1 and TcTR2)
Tcas_CX	GTGTGACCTGACCTGACC	
VRP440	GACAATCCGTCGAGCAGAGTT	TRAP (TcTR1aa)
Tcas_CXaa	GTGTAACCTGACCTAACC	

>pEX-A258-3xFLAG-TcTERT

TAATACGACTCACTATAGGGAGACCCAAGCTGGCTAGTTAAGCTTGGTACCGAGCTCGGATCCACCATGGACTACAAAGACCATGACGGTGATTATAAAGATC
ATGATATCGATTACAAGGATGACGATGACAAGGAATTCATGGTCCACTACTATCGCCTTTCGTTAAAATCCCGCCAGAAAGCTCCAAAAATAGTCAACTCAAA
ATACAACAGCATCTTAAACATTGCTCTAAAAAACTTCCGACTATGTAAAAAGCACAAGACCAAGAAACCCGTCCAAATTCTCGCACTTTTACAGGAAATCATT
CCAAAATCTTACTTCGGAACAACCACAAACCTCAAACGTTTCTACAAAGTCGTCGAAAAAATTCTAACCCAATCGTCATTCGAGTGCATCCATTTATCAGTCC
TCCATAAATGCTACGATTATGATGCAATTCCCTGGCTACAAAACGTCGAACCGAACCTCCGCCCTAAGCTATTGCTCAAACACAATTTATTCCTCCTCGACAA
CATAGTCAAACCTATAATCGCCTTTTACTACAAACCCATCAAAACACTGAACGGACACGAAATCAAATTCATCCGCAAAGAGGAGTACATTTCATTCGAGAGC
AAGGTGTTTCACAAATTGAAGAAAATGAAGTATTTGGTCGAGGTCCAAGACGAGGTGAAACCACGAGGTGTTTTGAACATAATCCCAAAACAAGACAACTTTC
GCGCAATCGTGAGCATATTCCCAGATTCGGCGCGAAAACCATTTTTTAAACTTTTAACGTCCAAAATCTACAAAGTGTTGGAAGAGAAATACAAAACAAGCGG
GTCTCTCTACACGTGCTGGTCGGAATTTACTCAAAAAACACAAGGGCAGATTTATGGCATCAAAGTTGATATTAGAGACGCGTACGGAAACGTCAAAATTCCA
GTTCTTTGTAAATTAATTCAGAGCATTCCGACACATTTGCTGGATTCGGAAAAGAAAAATTTTATTGTAGACCACATCAGCAACCAATTTGTGGCTTTTAGGC
GAAAGATTTACAAGTGGAACCACGGATTGCTACAAGGTGATCCCTTATCAGGCTGTTTGTGTGAACTCTACATGGCTTTTATGGACCGACTTTACTTTTCCAA
CTTGGACAAAGACGCTTTCATCCATCGAACCGTCGACGATTATTTCTTCTGCTCACCTCACCCTCACAAAGTCTACGACTTCGAATTGCTAATCAAAGGCGTC
TATCAAGTAAATCCCACCAAAACACGAACAAATTTACCAACCCATCGCCACCCACAAGACGAAATTCCCTATTGCGGAAAAATCTTCAATTTGACGACACGAC
AAGTGCGTACGCTATACAAACTGCCTCCGAATTACGAGATTCGACACAAATTCAAACTGTGGAATTTTAACAATCAAATCAGCGACGATAACCCGGCGAGATT
TCTCCAAAAAGCGATGGACTTTCCATTCATTTGTAACAGTTTCACGAAGTTTGAATTTAACACAGTTTTCAACGACCAAAGGACCGTTTTTGCGAATTTCTAC
GATGCTATGATTTGTGTCGCTTATAAGTTTGATGCAGCTATGATGGCATTAAGAACTAGTTTTTTGGTCAATGATTTTGGTTTTATTTGGTTAGTTTTGAGTT
CGACAGTTAGAGCATATGCCTCGAGGGCGTTTAAAAAAATCGTAACTTATAAAGGTGGCAAATATCGGAAAGTAACATTTCAGTGTTTGAAAAGTATCGCTTG
GCGGGCTTTTCTTGCTGTTCTTAAGCGAAGAACTGAAATTTATAAAGGGTTAATTGACAGAATTAAAAGCCGGGAAAAGTTGACTATGAAGTTTCATGATGGG
GAGGTGGATGCGAGTTATTTTTGTAAATTGCCGGAAAAGTTTAGATTTGTAAAAATCAACAGAAAGGCCTCAATTTAAGAATTCTAGCATAACCCCTTGGGGC
CTCTAAACGGGTCTTGAGGGGTTTTTTG

>pEX-A258-TcTR1

CCACCCCCAAATATTTCTTTCAAGAGAGGGCGCGTGAATCTACATTTTGCAAACACTTTGGTACCTTACAGGTTTATATCTTTCATAAAAACAAATTCACATC
GCTCTCGCATAGCAGAGCCCATCAGCATGGGTTTGTGAGCTTCACCCACCCAATTACTGATGACCTGACCTGACCATCTCATGTCTAATGGGGTTATTTTGTA
ATTACTACCTTTCTCCCCTCAAAAGAAAATAAATCAAATAAAAGCATATTATTTCTAGTTTCAAGTTATCTAACTTCAATCTTGCATTACCTGACAAAAACAC
ATTTCATATTTTTT

>pEX-A258-TcTR2
TTGCAATAGAGGGCGCACGGATCTAATATTTACTAACCCTCAGACTACCTAACGTGTTCATGTTTAGCAGAAAAGCATTTTTT
CATCGCTCTCACAGACACAGAGCCCATCAGCATGGTTCGTTCGTTCGTTCAATGTGCAGTTCACTGTCTGATGTGCTGACCT
GACCAACGCACGTCTTATGGGGTTCTTTTGTAATTGCTACATTTCTCCCCTCAAAAGAAAATAAAATAATCAAAGCATATTCC
ACTTTTATTT

References

1. Fajkus P, Kilar A, Nelson ADL, Hola M, Peska V, Goffova I, Fojtova M, Zachova D, Fulneckova J, Fajkus J: **Evolution of plant telomerase RNAs: farther to the past, deeper to the roots.** *Nucleic Acids Res* 2021, **49:**7680-7694.
2. Bolger AM, Lohse M, Usadel B: **Trimmomatic: a flexible trimmer for Illumina sequence data.** *Bioinformatics* 2014, **30:**2114-2120.
3. Kopylova E, Noe L, Touzet H: **SortMeRNA: fast and accurate filtering of ribosomal RNAs in metatranscriptomic data.** *Bioinformatics* 2012, **28:**3211-3217.
4. Dobin A, Davis CA, Schlesinger F, Drenkow J, Zaleski C, Jha S, Batut P, Chaisson M, Gingeras TR: **STAR: ultrafast universal RNA-seq aligner.** *Bioinformatics* 2013, **29:**15-21.
5. Li B, Dewey CN: **RSEM: accurate transcript quantification from RNA-Seq data with or without a reference genome.** *Bmc Bioinformatics* 2011, **12**.
6. Schuller AP, Harkisheimer MJ, Skordalakes E: **In vitro reconstitution of the active T. castaneum telomerase.** *J Vis Exp* 2011**:**e2799.
7. Fajkus P, Adamik M, Nelson ADL, Kilar AM, Franek M, Bubenik M, Frydrychova RC, Votavova A, Sykorova E, Fajkus J, Peska V: **Telomerase RNA in Hymenoptera (Insecta) switched to plant/ciliate-like biogenesis.** *Nucleic Acids Res* 2023, **51:**420-433.

A *Tribolium castaneum* (Tenebrionoidea)

```
                                                                                                                                              TSS
                                                                                                                                               ↓
U1     TAGTTATGAGAGAGGCGAATAGATGCCGCTGCAATCTTGTTCCTACGAGTTTTAGCCGCCCCTTAGGTATTGAAGACTGATACCAGGTGCTTGCCAAAGC
U2     AAGTACGGTAAGTTTCTACGACATGGCGCCACTATCTGCTGTTCACGATGCTTAACGTACAGTTTGACAACAAATAGGTGTTTCGGAGAATTGTTCGGCA
U3     TCGTTGTGATAACCCCTAGTAGATGTCGTCCTTAGTTGTTACTACCGAACGTTTCTGGTTCTGTGCTACACAACAACGATTGGTTTCGTTGGAAAAATCA
U4     CGGTTGCTTGGTTGGTTCGTAGATGGCGATAGTATGTACAAATTCCGGTTACCTCTCTGCCCAAGTCACGATAATACTTCACTGGAGCAAAGCTTTCGTC
U5     GTAGTAGGAAGTGTTCCAATAGAGGGCGAAACTTGTAGCGTTTCGCTCGTTTGGGACGTCCCTCTCGTGATAAATTCCTACTGAGAGTGGTTTTCGTCAC
U11    TACTTCCAGCGCACGACCCGAGATAGCGCTCGCTGCTTCGTCTCACGACTAGAATGGGGACGCTCTCACGTTTATACACGAGGAGCGACCAAGCGAGTTC
                                 <Pol II PSE>
Cons.  NNVTTVBDWNRNNBKHYMVKAGATGBCGMYMSHWKYTKBKWYTYMCGVNBNBNWNNSKNHCSNHNBNWMRYWAADACNNNWNKNDNVNNNWKNYNNNNNM

TcTR1  GTATACTTTTGGCTTTCAAGAGAGGGCGCGTGAATCTACATTTTGCAAACACTTTGGTACCTTACAGGTTTATATCTTTCATAAAAACAAATTCACATCG
TcTR2  AAAACCATAAGTCTTGCAATAGAGGGCGCACGGATCTAATATTTACTAACCCTCAGACTACCTAACGTGTTCATGTTTAGCAGAAAAGCATTTTTTCATC

Cons.  TTDDWTVDVNDHDDADVDDDHDTTDWAANKHBGTTACATCTCACTACYGBTHVHCGTHCVVNBDTAVBHWTATATACRADTHBNCDVDGTTNDNBBDDTG
                                                 <Pol III PSE>                           <TATA box>
7SK    TCGTTTGGAAAAAAAAGTGTCATTATTTGGAGGTTACATCTCACTACTGCTCGCCGTTCCCCTATAACTATATATACGAATCCCCTCTCTTATTTCGTTG
MRP    TTAAAAAGCCGTAAATAGTGTTAATTAAATTCGTTACATCTCACTACCGGTTATCGTCCGCTCGTACGCTTATATACGAGTATTCGAAGGTCACTCAGTG
U6     ATTGATCAGGACGGCGCTGAAATTTAAACGCGGTTACATCTCACTACTGCTACACGGACAGGGTTGAGCTTAAATACAATTTGGAAAGGTGGGACTTTTG
U6atac TTAATTGTCTTTTTATGAATTGTTGAAATTTTGTTATATCTCACTAACGTTAGACGTCCGAATGTAGTAATATATACAGTGAGACGGGGTTTGGGGAATT
```

B

Pediacus dermestoides (Cucujidae)

```
                                                                                                                                           TSS
                                                                                                                                         (±3nt)
                                                                                                                                            ↓
cons.  RHNRNBVNTAGATGGBGVWRKNMNTKDWADBTTGCKGYTKNWKWKYHKWNKNWWWKNKDTAWAWWBDBVABKSRRDYWDBNDDNMRKNWHNNTBNNNHBY

TR     AATATGATTAGATGGCGCAATCCTTGCAAA-CCGCGGTTTCTAAGTCGAATAGTTGAGATATATTTCAAAGGCTAACAACAGATAACGTCTTTTTGGACC
U1     ACGGGGCATAGATGGCGCATGTAATTATAGCTTGCTGTTGGTTGTTTTAGTATTTGCGTTAAATAGTGGACGTGGAGCGGGAGAAAGCATACTTACCTGG
U2     ATCTCTCCTAGATGGTGCTGTAATTTGGATGTTGCTTCTTAGTTTCATGTTAAATTTGATATAAACGGACTGGAAATTTTATTCAGTATCGCTTCTCGGC
U3     GCTACCACTAGATGGGAGTGGACGTTGTAAGTTGCGGTTATAGAGTCGTCGGATTGTGGTAAATAGGCGAGTGAAACAAGTGAACGGCAAGATCATACTT
U4     AACGACAAGAGATGGCGCTGTTGCTTTTAAGTTGCTGCTTCTGAGTGGTTTTAAAGTTATATGATAATCAGTGGATCTGGATGGCGTTGTGATGAATCCT
U5     GTTACTGGTAGATGGCGCTGTGCTTATAGG-TTGCGGTTGCAGTTCAATCAGTACAGAATAAATACTCCACGGAGGCACTGATTCGGTACTCTGGTTTCC
U11    TTAACCGATAGATGGCGACAAACTTGATATTTTGCCGTTGAATAACTTACGCTTATGTATAAATGTATAATACATATATCCTTTAGTTTACGACGACATT
```

Cantharis rufa (Elateroidea)

```
Cons.  WMMNWMWWHNWDKDHWYSATAGATGGCRYYAVNRNKRNNWKYYSYNWRTNDANWWTHHDBNWVNNNDVNABWWAAMNSNNARNRMNWDHNMRWMWNDNHW

TR     AAGAACGTCGAAGTCAAGATAGATGGCACTAACGAAAATTGTTGCTAATGGTATTTTTGCATATGATTTATTTAAATATCAGAAAATTCGCATTTGGTAT
U1     CCAATATATTTAGACATCATAGATGGCGTAACAAATAACTTCCGCCAGTCTATTATTTGTAAAACTACCACAAAACTCCAAGTAAAACCTCGAAAAGCAT
U2     TCCAACTTCATGAGATTAATAGATGGCGTTACCTCGGCTTGTTGTATATCGAAATTTCGTGAAGAGTAAATATAAATCTCACCGCGAGATCGAATAAGTA
U3     TACCTTAAAATTGTTTCGATAGATGGCGTTACCGTTAAAAGTACCTAATCAAGTTAATGGAACCACTGCACTTAAGCGAAAAAAAAAATTAAAAAAATCA
U4     TTCGTATATCTGTTTACGATAGATGGCAGCACGAGTGTAAATTACGTGTAAATAATTAATCAGAAGTAGAGAAAAAAGGTAGCGATTTCACAAATCTTTA
U5     AAAAAAAAAAATTTATTCATAGATGGCGCTACGGATTCACTCCCTAGTAATATTTTCTGTTCCAGTTCACATAAACGCTGAAATCCAAAAAATCTTTACT
U11    TAATACATTTCGTTTGCGATAGATGGCGTCAGTGTGGGGTTCTGCTAGTTAACATTTTTTATGATGGGCAGATAAACGCATGGATTTGTCGGGCAAATTC
```

Agonum fuliginosum (Carabidae)

```
Cons.  NNNWWNDVHMNHWRRWDRWRHWDBNRNKHVWHNYHTRDYTCTCCGTNCDHNTTACCWKYGYRVRWWKNDHRDNMYRNNNNNRRHKDSNMSNYWBYRKNRN

TR     AAGTATAATTTCAAATAAAATAAAAAATTCTATTTTTTCTCTCCGTCCTTCATACCTACGCACATTGTTTGAATGATAGTTCTAGGCACGTTCTCGTTGT
U1     AGAAGTATAAAATAGAAAAAGTGTAGGTGGTTCTCTGGTTCTCCGTGCATGTTACCAGCGCGAGTAGAGTGATATAGGCCTGGCAGGCCGCTAACAAAGC
U2     TCCAAAAATCGTCTATCCAAATTTTGATTGTTATAAAATTCTCCGTACATATTACCTGTGCAATTAGATCATACCGAGTAAAATTTGTCGGTACCAGTAT
U3     CTTTTTGCTCCATAGATGTCTTTGGGCGCTACTCATACTTCTCCGTTCGTTTTACCAGTGCGGAAATGGTAACACGGGTCTGGCGAGCCCAATCAGGCAA
U4     CCGTTGTGTAACAGATGGTGCTGGGTGTCAGCACTTAGCTCTCAGTTTACTTTACCATCGCAGGATTCATAGGATGGTATGGGTGGGAAGGTATCGTCAG
U5     GATATAAGTAAATGTATAAAATACGATGAAAATTTTATCTCTCCGTCCATTTTACCATCGTCCATGTAGACTACTACCTGAAAATAGGACTCAGTCGGTA
U11    ACTTTCACCCATAGATGACGCAGCCAGTACACGACTGACTCTCCGTTCAAGTAGCCTTTGTGAGTTTTATAGCACGGCTCCGGTTTCAGCTCTGTATCAT
```

■ 100% similarity ■ 80-99% similarity

Supplementary Figure S1. Comparison of TR and snRNA promoter regions. (**A**) Aligned upstream regions of transcription start sites (TSS) of representative type 3 snRNA promoters transcribed by RNA polymerase II or III. Conserved nucleotides are highlighted in the consensus sequence (Cons.). These regions were compared with the corresponding upstream TSS regions of both Tribolium castaneum TR paralogs, TcTR1 and TcTR2. (**B**) Orthogonal comparison restricted to RNA polymerase II–dependent snRNA promoters and the corresponding upstream promoter regions of TRs from evolutionarily more distant beetle taxa.

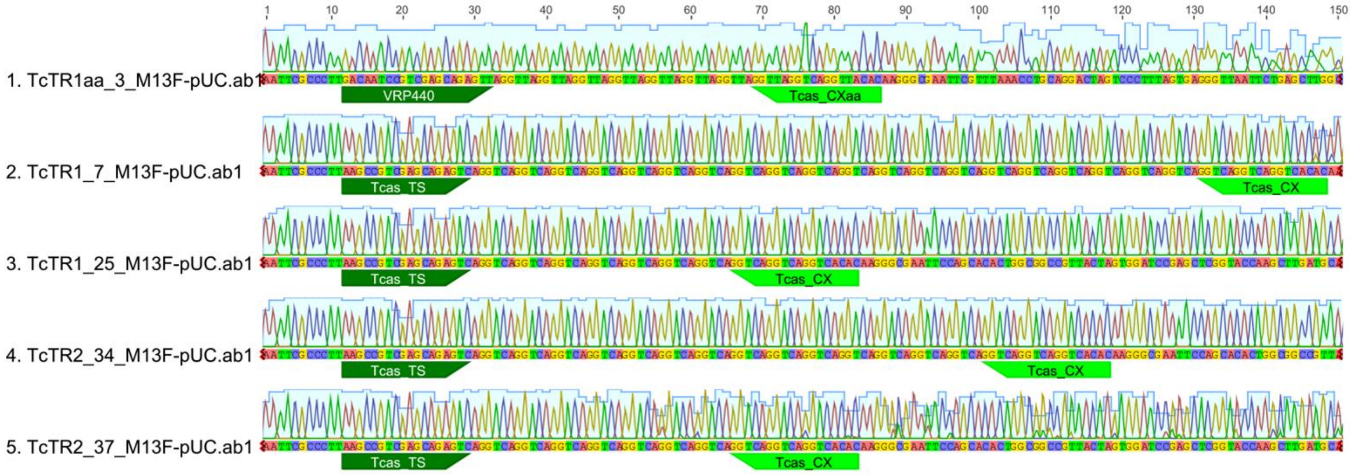

Supplementary Figure S2. TRAP product sequencing. TRAP reaction products generated using TcTR1aa, TcTR1, and TcTR2 RNA templates were cloned. Clones containing longer insert products were selected by colony screening and submitted for Sanger sequencing to verify the identity of telomerase extension products.

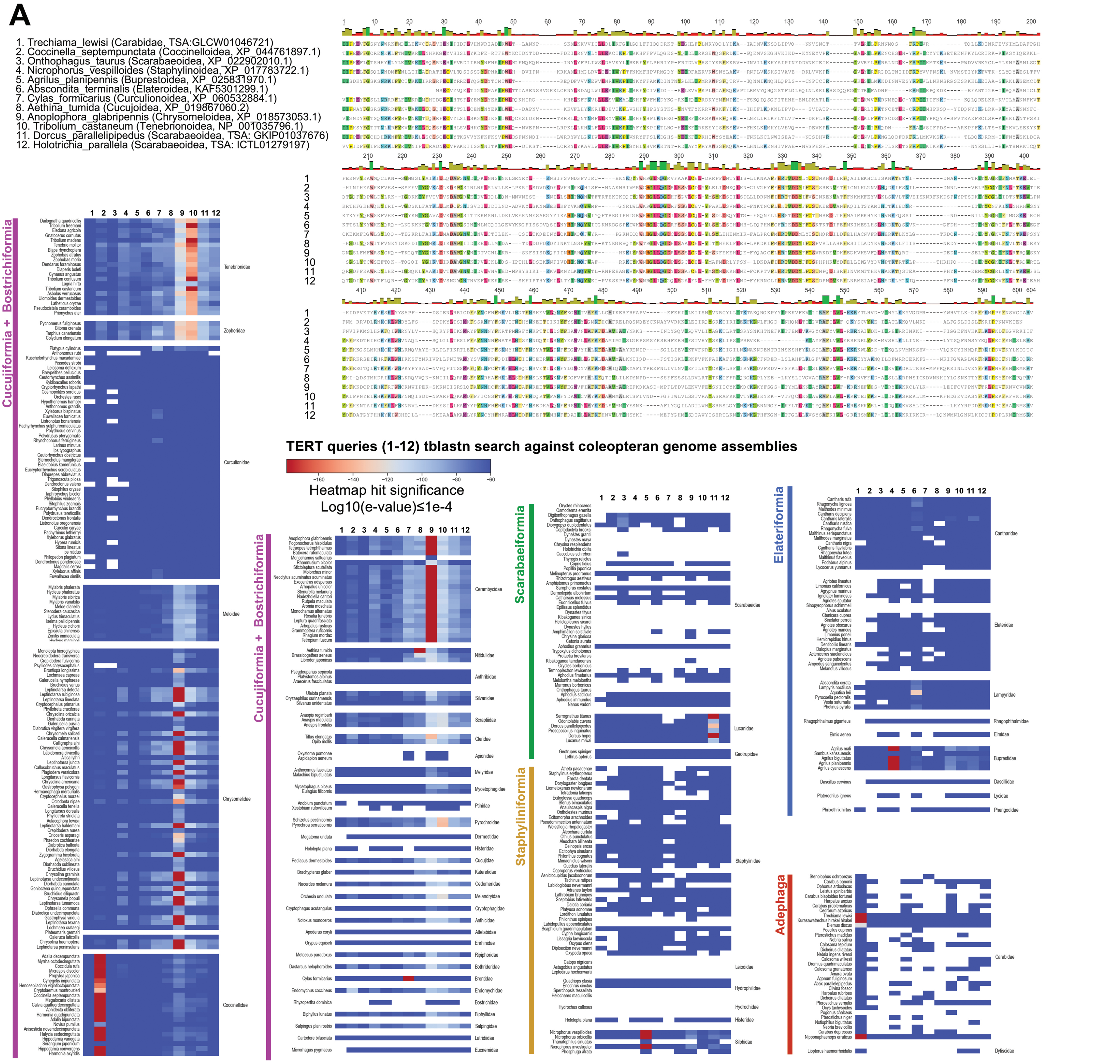

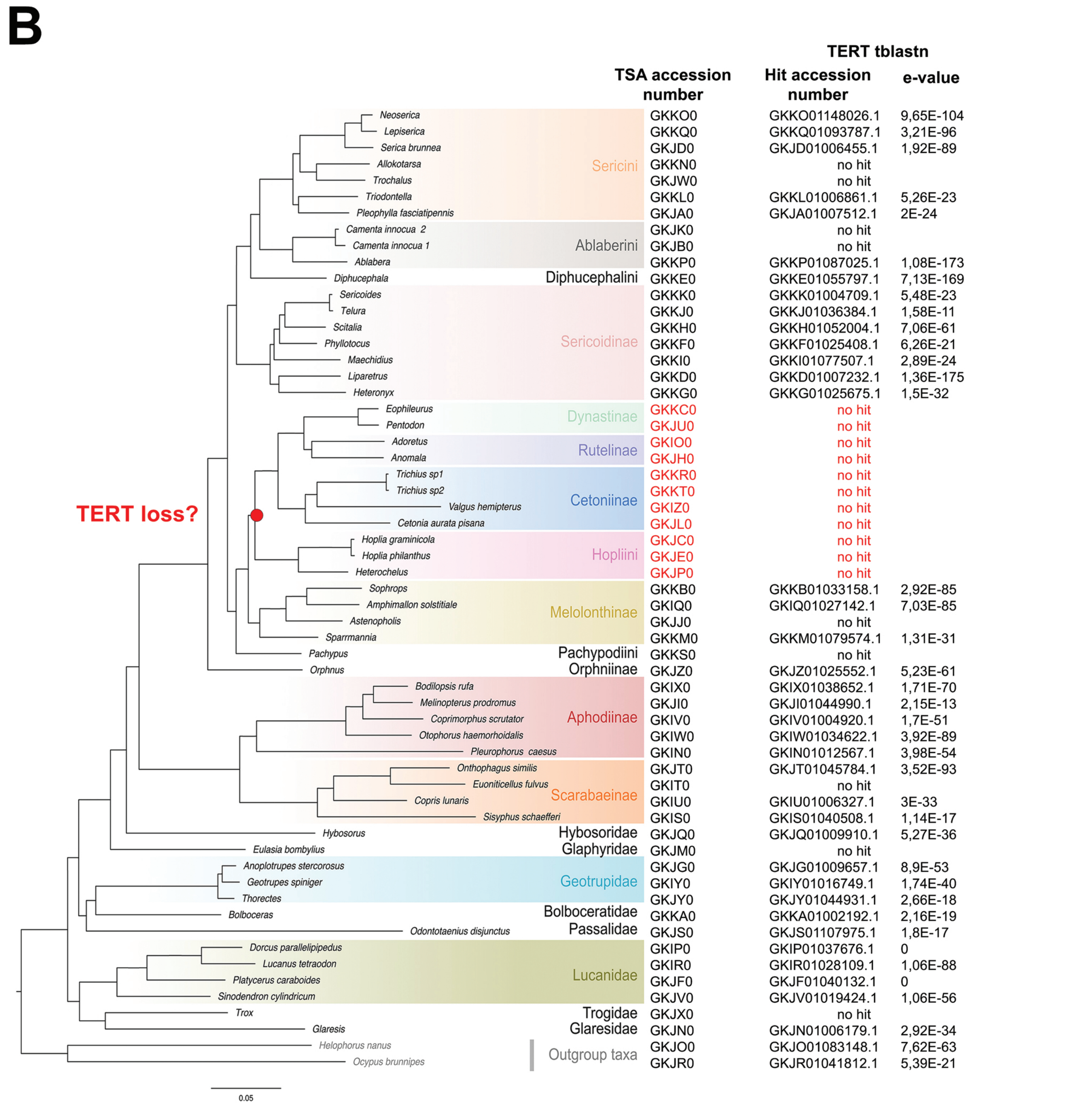

Supplementary Figure S3. Search for TERT gene. **(A)** A set of 12 representative TERT protein sequences spanning major coleopteran clades was aligned and used as independent queries in tblastn searches against assembled beetle genomes. Significant hits (E-value ≤ 1e−4) were visualized as heatmaps for each of the 12 queries. This screening revealed a frequent absence of detectable TERT homologs in **Scarabaeoidea**, despite the large number of available genome assemblies for this clade.
(B) TERT detection within Scarabaeoidea was further examined using available transcriptomic (TSA) data, as indicated in the next to phylogeny tree, and three Scarabaeoidea-derived TERT queries (queries 3, 11, and 12). In addition, PacBio datasets from cetoniine genomes with available T2T assemblies (*Cetonia aurata, Protaetia brevitarsis*) were screened. No TERT homologs were recovered in the lineages Dynastinae, Rutelinae, Cetoniinae, and Hopliini, even when using the TERT CDS from the closely related Amphimallon (Melolonthidae) as a query. Together with the results from genome-based screening, T2T assemblies, and TRFi analyses, these data indicate a systematic absence of detectable TERT under the applied search conditions and suggest that telomere maintenance in these lineages may rely on a non-canonical, telomerase-independent mechanism.

The phylogenetic tree and corresponding TSA data were adapted from Dietz et al. (2023), Systematic Entomology, https://doi.org/10.1111/syen.12602

Supplementary Figure S4. TeloSearchLR analysis (doi: 10.1093/g3journal/jkaf062) of the *Cetonia aurata* long-read whole-genome dataset (SRA accession: ERR10753927). Candidate repeat sequences showing terminal enrichment are presented. These repeats were subsequently compared with chromosome ends of the T2T genome assembly (GCA_949128085.1), where corresponding matches are highlighted in yellow.

>repeat046
TTTTTTTTTTTTTTTTTTTTTTTTTTTTTTTTTTTTTTTTTTTTTTTTTTT

>repeat098
TGGGGGTGGGTCGTTTTCGGGTCAAATGGGCCTGGTGGGGGTGGGTCGAATCCGGGCGAAACTCCGGGGGTGGGTCATTTCCGGGTCTGATGGGTTTGG
>repeat059
TGGGGGTGGGGCATATCCGGGTCTCACGGGTTCGG
>repeat074
TGGGGGTGGGTCGTTCCTGGGCCAAAGTCCGGGGGTGGGTGGTTTTTTGGGCCGACGGGCCTGG
>repeat090
TGGGGGTGGGTCGTTCGGGGGGCAAGTTCCGGGGGTGGGTCGTTTTTTGGGCCGACGGGCCTGG
>repeat081
TGGGGGTGGGTCGTTTCCGGGTCAAATGGGCCTGG

>repeat079
GTGATGATGATGGTGGTGATATG

>repeat082
CAACAACTGACCATTTAAGGTGCAACAACTGACCATTTAAGGTGCAACAACTGACCATTTAAGGTG

>repeat004
GCATTGTTTTTCAGGGGTGAGAAACCGTTGCTAGTTTGATCTTTAGTGTTACTGTGAGATTGCTGAACTTATAAGTTTGAAATCTTTATTGAC
>repeat014
GCAGTGTTTTTCAGGGGTGAGAAACCGTTGCTAGTTTGATTTTTAGTGTTACTGTGAGATTGCTGAACTTATAAGTTTGAAATTTTTATTGAC
>repeat017
GCAGTGTTTTTCAGGGGTGAGAAACTGTTGCTAGTTTGATTTTTAGTGTTACTGTGAGATTGCTGAACTTATAAGTTTGAAATCTTTATTGAC
>repeat023
GCATTGTTTTTCAGGGGTGAGAAACCGTTGCTAGTTTGATCTTTAGTGTTATTGTGAGATTGCTGAACTTATAAGTTTGAAATCTTTATTGAC
>repeat027
GCATTGTTTTTCAGGGGTGAGAAACTGTTGCTAGTTTGATTTTTAGTGTTACTGTGAGATTGCTGAACTTATAAGTTTGAAATCTTTATTGAC
>repeat029
GCAGTGTTTTTCAGGGGTGAGAAACCGTTGCTAGTTTGATCTTTAGTGTTACTGTGAGATTGCTGAACTTATAAGTTTGAAATTTTTATTGAC
>repeat032
GCATTGTTTTTCAGGGGTGAGAAACCGTTGCTAGTTTGATTTTTAGTGTTATTGTGAGATTGCTGAACTTATAAGTTTGAAATCTTTATTGAC
>repeat036
GCAGTGTTTTTCAGGGGTGAGAAACTGTTGCTAGTTTGATTTTTAGTGTTATTGTGAGATTGCTGAACTTATAAGTTTGAAATTTTTATTGAT
>repeat039
GCATTGTTTTTCAGGGGTGAGAAACCGTTGCTAGTTTGATTTTTAGTGTTATTGTGAGATTGCTGAACTTATAAGTTTGTCCTTTTTATTGAC
>repeat043
GCAGTGTTTTTCAGGGGTGAGAAACTGTTGCTAGTTTGATTTTTAGTGTTATTGTGAGATTACTGAACTTATAAGTTTGAAATTTTTATTGAT
>repeat053
GCAGTGTTTTTCAGGGGTGAGAAACCGTTGCTAGTTTGATTTTTAGTGTTATTGAGAGATTGCTGAACTTATAAGTTTGTCCTTTTTATTGAC
>repeat056
GCAGTGTTTTTCAGGGGTGAGAAACTGTTGCTAGTTTGATTTTTAGTGTTACTGTAAGATTACTGAACTTATAAGTTTGTCCTTTTTATTGAC
>repeat058
GCAGTGTTTTTCAGGGGTGAGAAACCGTTGCTAGTTTGACCTTTAGTTTTACTGTGAGATTGCTGAACTTATAAGTTTGAAATCTTTATTGAC
>repeat069
GCAGTGTTTTTCAGGGGTGAGAAACCGTTGCTAGTTTGATCTTTAGTGTTATTGAGAGATTGCTGAACTTATAAGTTTGAAATCTTTATTGAC
>repeat072
GCAGTTTTTTTCAGAGGTGAGAAACCGTTGCTAGTTTGATCTTTAGTGTTACTATGAGATTGCTGAACTTATAAGTTTGAAATCTTTATTGAC
>repeat075
GCAGTGTTTTTCAGGGGTGAGAAACCGTTGCTAGTTTGGTTTTTAGTGGTACTATGAGATTGCTGAACTTATAAGTTTGTCCTTTTTATTGAC
>repeat080
GCAGTTTTTTTCAGGGGCGAGAAACCGTTGCTAGTTTGATTTTTAGTGTTACTGTGAGATTGCTGAACTTATAAGTTTGAAATCTTTATTGAC
>repeat093
GCAGTGTTTTTCAGGGGTGAGAAACTGTTGCTAGTTTGATTTTTAGTGTTATTGAGAGATTACTGAACTTATAAGTTTGAAATCTTTATTGAC
>repeat100
GCAGTGTTTTTCAGGGGTGAGAAACCGTTGCTAGTTTGACCTTTAGTGTTATTGAGAGATTGTTGAACTTATAAGTTTGTCCTTTTTATTGAC

T2T genome assembly (GCA_949128085.1)

>repeat098
TGGGGGTGGGTCGTTTTCGGGTCAAAT
GGGCCTGGTGGGGGTGGGTCGAATC
CGGGCGAAACTCCGGGGGTGGGTCA
TTTCCGGGTCTGATGGGTTTGG

12 of 22 chr. ends ~ repeat098

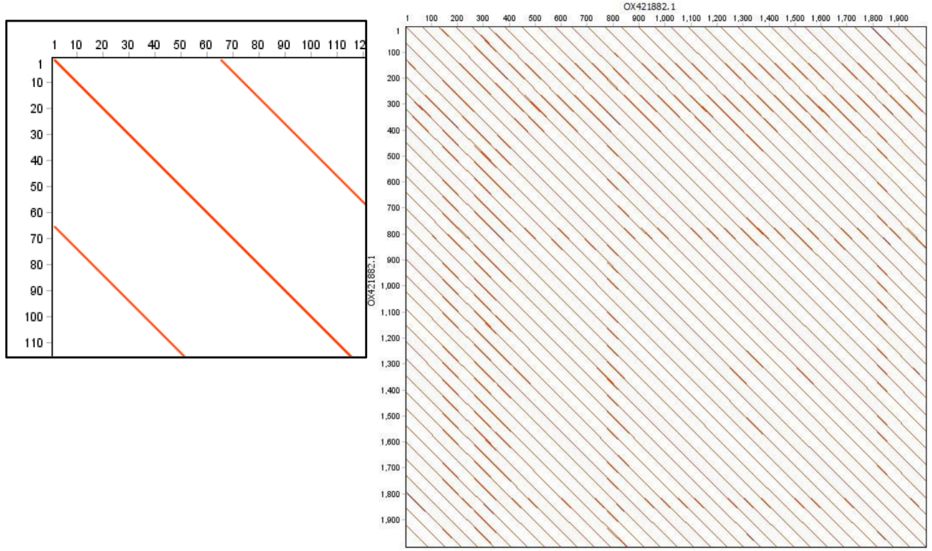

Example dotplot of chr_1_left end (~64nt satellite)

Repeat059 (occupancyMode; k21; K50; t1000; n6000 ERR10753927)

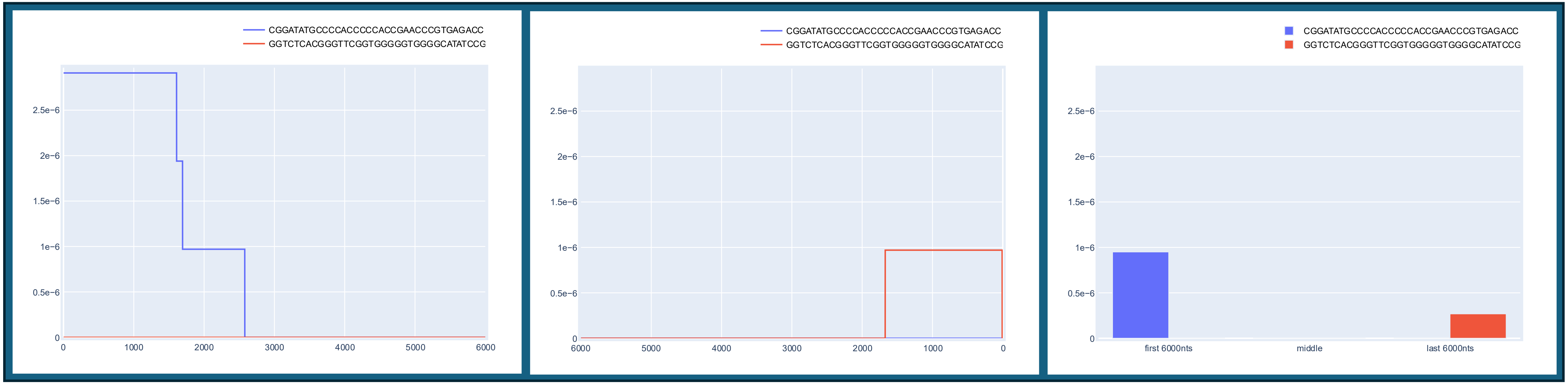

repeat pattern:
CGGATATGCCCCACCCCCACCGAACCCGTGAGACC
reverse complement:
GGTCTCACGGGTTCGGTGGGGGTGGGGCATATCCG

Repeat079 (occupancyMode; k21; K50; t1000; n6000 ERR10753927)

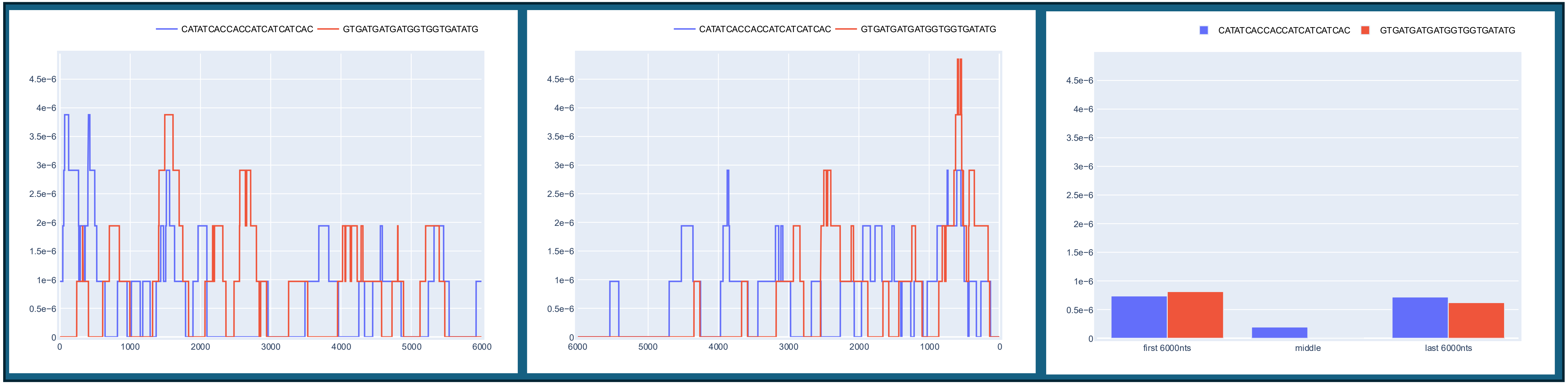

repeat pattern:
CATATCACCACCATCATCATCAC
reverse complement:
GTGATGATGATGGTGGTGATATG

Repeat081 (occupancyMode; k21; K50; t1000; n6000 ERR10753927)

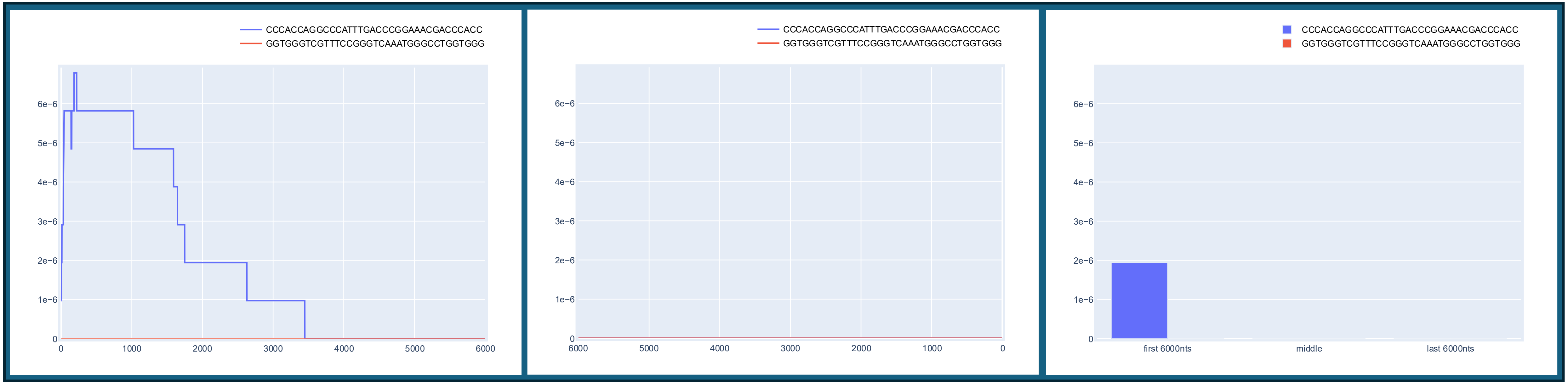

repeat pattern:
CCCACCAGGCCCATTTGACCCGGAAACGACCCACC
reverse complement:
GGTGGGTCGTTTCCGGGTCAAATGGGCCTGGTGGG

Repeat004 (occupancyMode; k51; K100; t1000; n6000 ERR10753927)

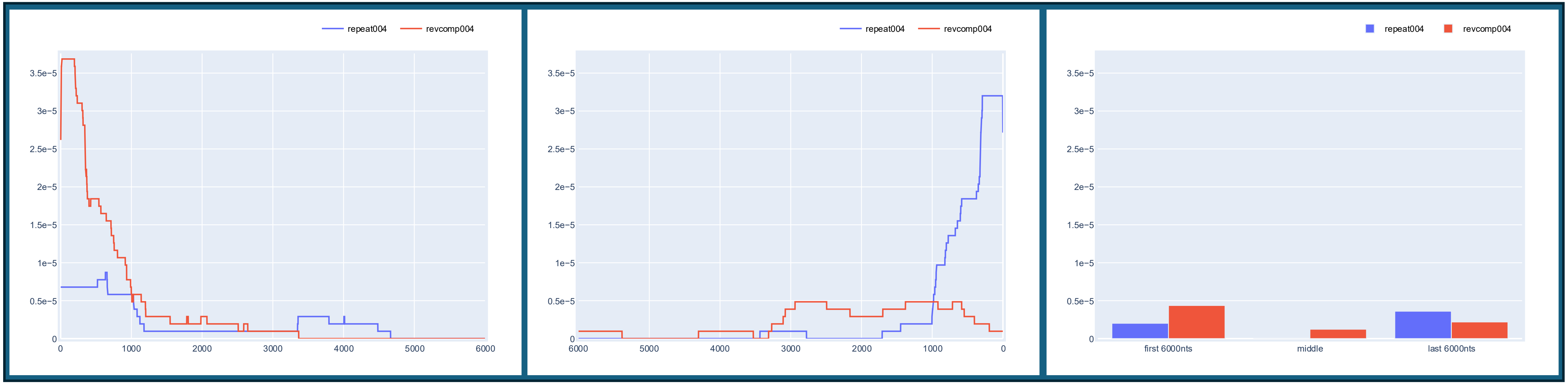

repeat pattern:
GCATTGTTTTTCAGGGGTGAGAAACCGTTGCTAGTTTGATCTTT
AGTGTTACTGTGAGATTGCTGAACTTATAAGTTTGAAATCTTTATT
GAC

reverse complement:
GTCAATAAAGATTTCAAACTTATAAGTTCAGCAATCTCACAGTA
ACACTAAAGATCAAACTAGCAACGGTTTCTCACCCCTGAAAA
ACAATGC

Repeat014 (occupancyMode; k51; K100; t1000; n6000 ERR10753927)

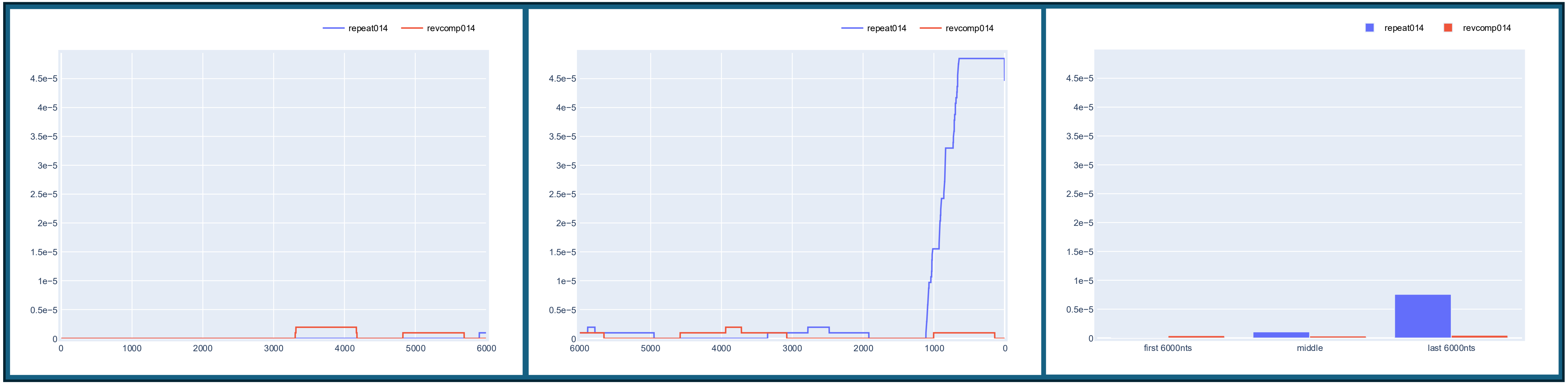

repeat pattern:
ACGGTTTCTCACCCCTGAAAAACACTGCGTCAATAAAAATTTC
AAACTTATAAGTTCAGCAATCTCACAGTAACACTAAAAATCAAA
CTAGCA

reverse complement:
TGCTAGTTTGATTTTTAGTGTTACTGTGAGATTGCTGAACTTATA
AGTTTGAAATTTTTATTGACGCAGTGTTTTTCAGGGGTGAGAAA
CCGT

Repeat017 (occupancyMode; k51; K100; t1000; n6000 ERR10753927)

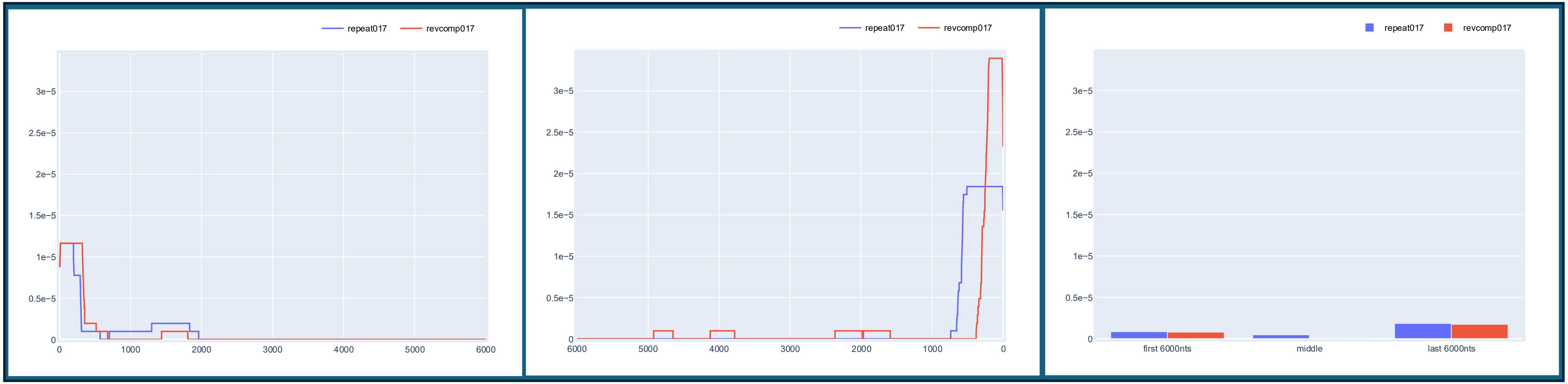

repeat pattern:
ACAGTTTCTCACCCCTGAAAAACACTGCGTCAATAAAGATTTCA
AACTTATAAGTTCAGCAATCTCACAGTAACACTAAAAATCAAAC
TAGCA

reverse complement:
TGCTAGTTTGATTTTTAGTGTTACTGTGAGATTGCTGAACTTATAA
GTTTGAAATCTTTATTGACGCAGTGTTTTTCAGGGGTGAGAAAC
TGT

Repeat023 (occupancyMode; k51; K100; t1000; n6000 ERR10753927)

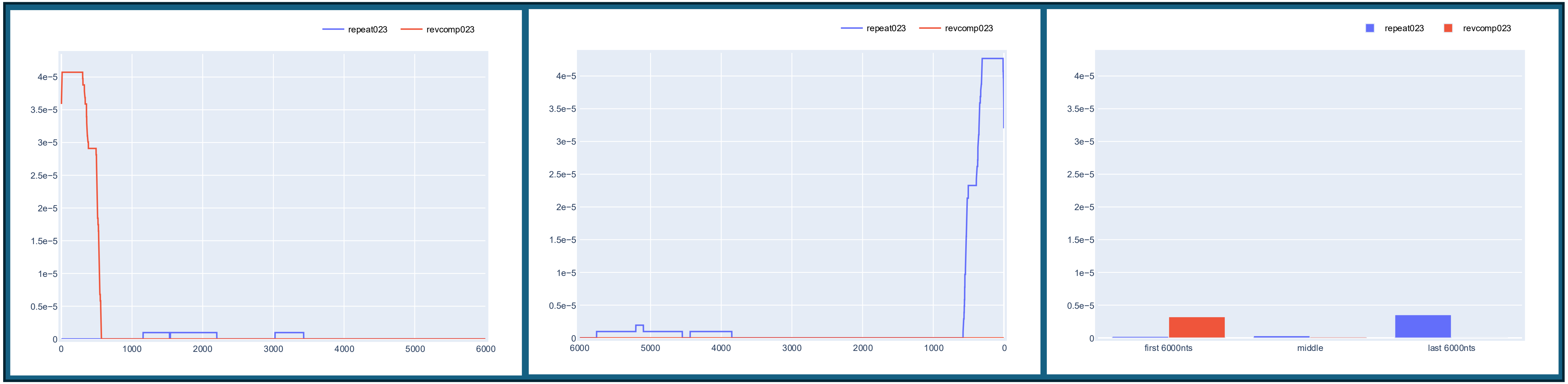

repeat pattern:
TAAGTTCAGCAATCTCACAATAACACTAAAGATCAAACTAGCAA
CGGTTTCTCACCCCTGAAAAACAATGCGTCAATAAAGATTTCA
AACTTA
reverse complement:
TAAGTTTGAAATCTTTATTGACGCATTGTTTTTCAGGGGTGAGAA
ACCGTTGCTAGTTTGATCTTTAGTGTTATTGTGAGATTGCTGAAC
TTA

Repeat27 (occupancyMode; k51; K100; t1000; n6000 ERR10753927)

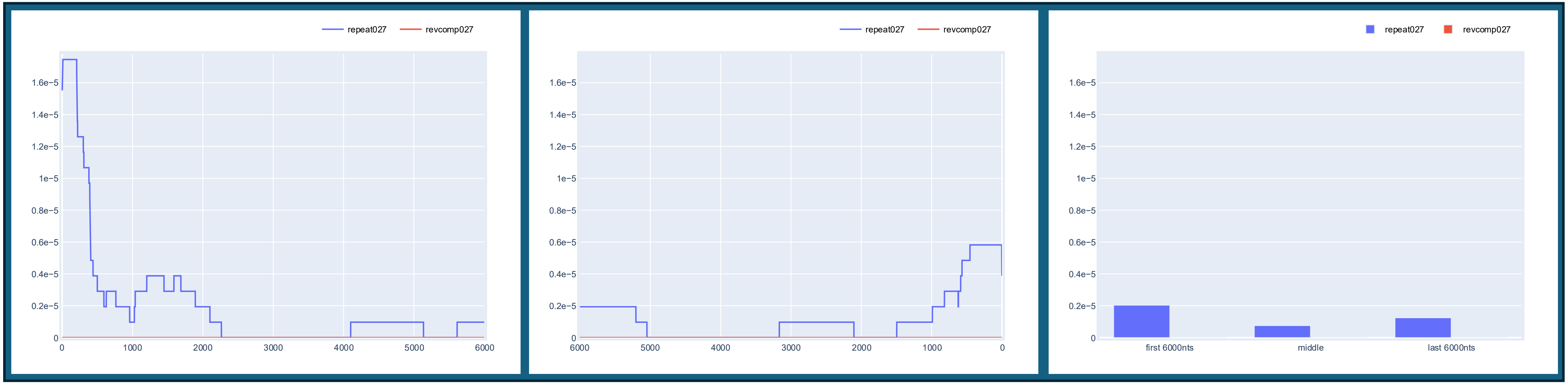

repeat pattern:
ACAGTTTCTCACCCCTGAAAAACAATGCGTCAATAAAGATTTC
AAACTTATAAGTTCAGCAATCTCACAGTAACACTAAAAATCAAA
CTAGCA

reverse complement:
TGCTAGTTTGATTTTTAGTGTTACTGTGAGATTGCTGAACTTATAA
GTTTGAAATCTTTATTGACGCATTGTTTTTCAGGGGTGAGAAACT
GT

Repeat029 (occupancyMode; k51; K100; t1000; n6000 ERR10753927)

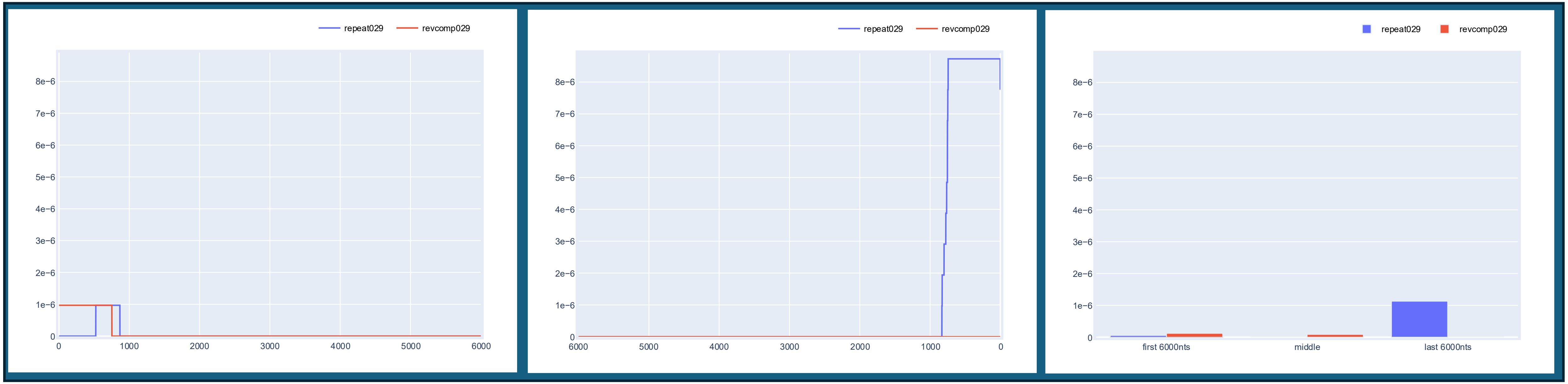

repeat pattern:
ACGGTTTCTCACCCCTGAAAAACACTGCGTCAATAAAAATTTC
AAACTTATAAGTTCAGCAATCTCACAGTAACACTAAAGATCAAA
CTAGCA

reverse complement:
TGCTAGTTTGATCTTTAGTGTTACTGTGAGATTGCTGAACTTATAA
GTTTGAAATTTTTATTGACGCAGTGTTTTTCAGGGGTGAGAAAC
CGT

Repeat032 (occupancyMode; k51; K100; t1000; n6000 ERR10753927)

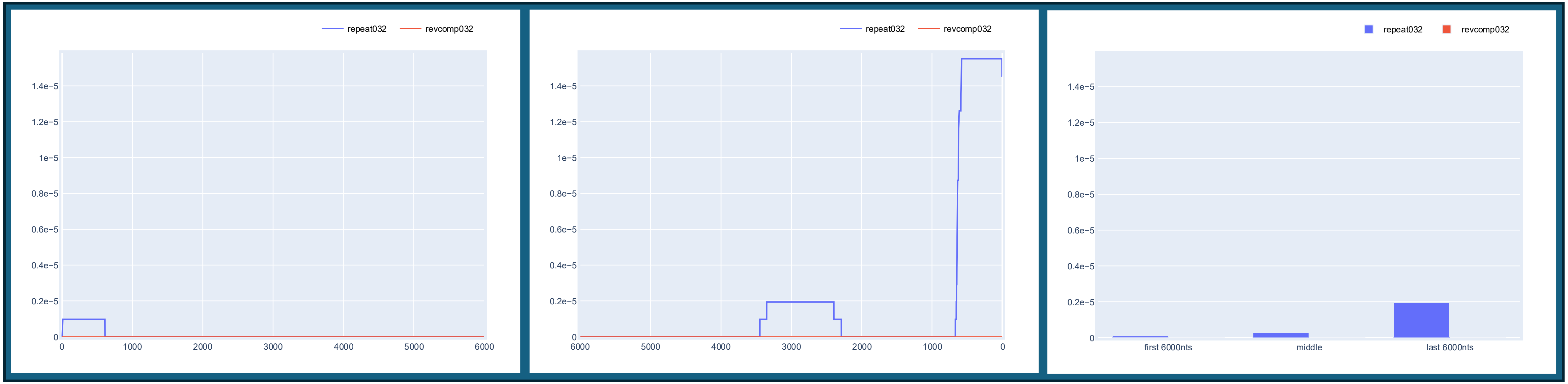

repeat pattern:
TAAGTTCAGCAATCTCACAATAACACTAAAAATCAAACTAGCAA
CGGTTTCTCACCCCTGAAAAACAATGCGTCAATAAAGATTTCA
AACTTA

reverse complement:
TAAGTTTGAAATCTTTATTGACGCATTGTTTTTCAGGGGTGAGAA
ACCGTTGCTAGTTTGATTTTTAGTGTTATTGTGAGATTGCTGAACT
TA

Repeat036 (occupancyMode; k51; K100; t1000; n6000 ERR10753927)

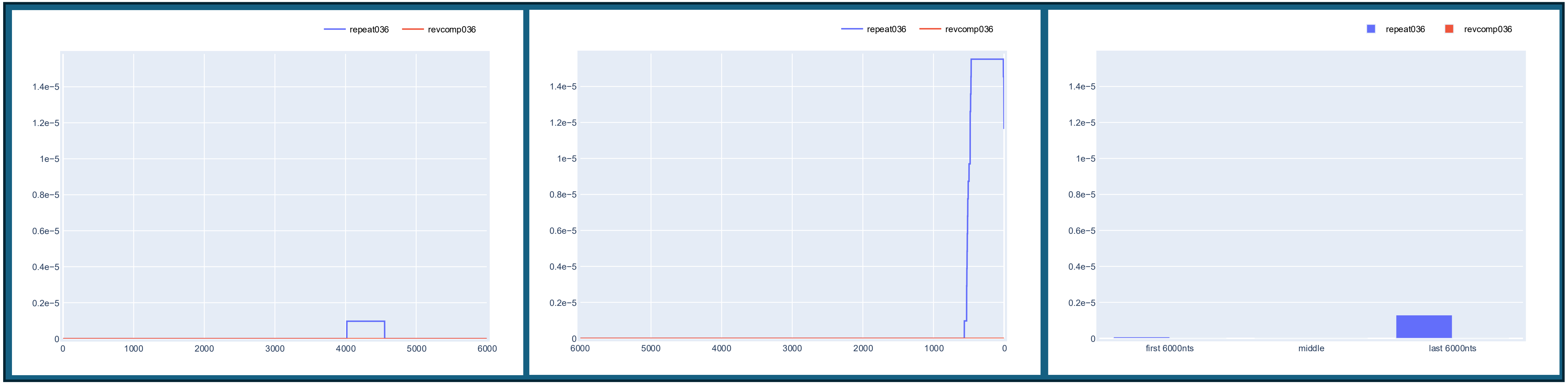

repeat pattern:
ACAGTTTCTCACCCCTGAAAAACACTGCATCAATAAAAATTTCA
AACTTATAAGTTCAGCAATCTCACAATAACACTAAAAATCAAAC
TAGCA

reverse complement:
TGCTAGTTTGATTTTTAGTGTTATTGTGAGATTGCTGAACTTATAA
GTTTGAAATTTTTATTGATGCAGTGTTTTTCAGGGGTGAGAAACT
GT

Repeat039 (occupancyMode; k51; K100; t1000; n6000 ERR10753927)

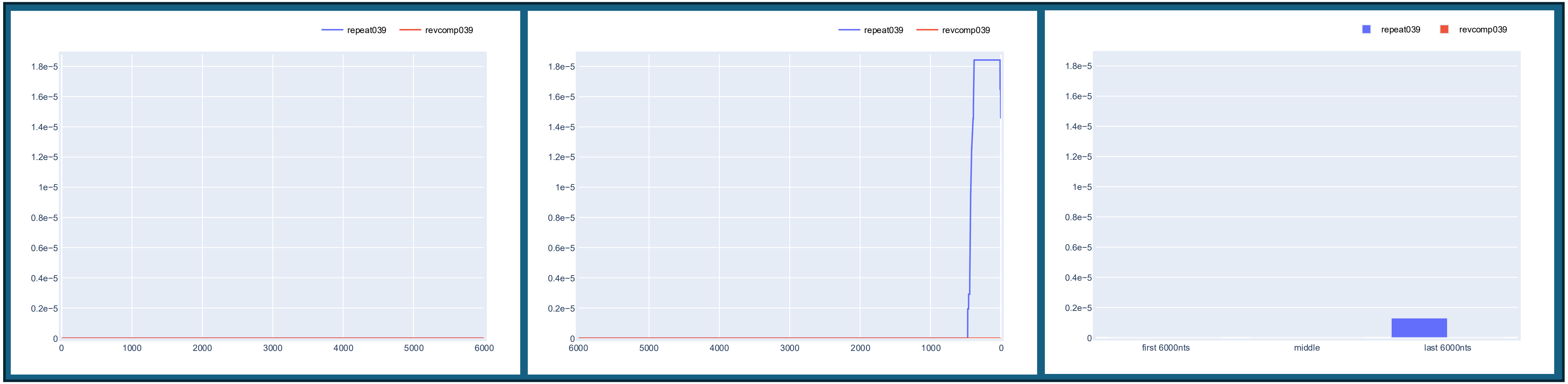

repeat pattern:
TAAGTTCAGCAATCTCACAATAACACTAAAAATCAAACTAGCAA
CGGTTTCTCACCCCTGAAAAACAATGCGTCAATAAAAAGGAC
AAACTTA

reverse complement:
TAAGTTTGTCCTTTTTATTGACGCATTGTTTTTCAGGGGTGAGAA
ACCGTTGCTAGTTTGATTTTTAGTGTTATTGTGAGATTGCTGAACT
TA

Repeat043 (occupancyMode; k51; K100; t1000; n6000 ERR10753927)

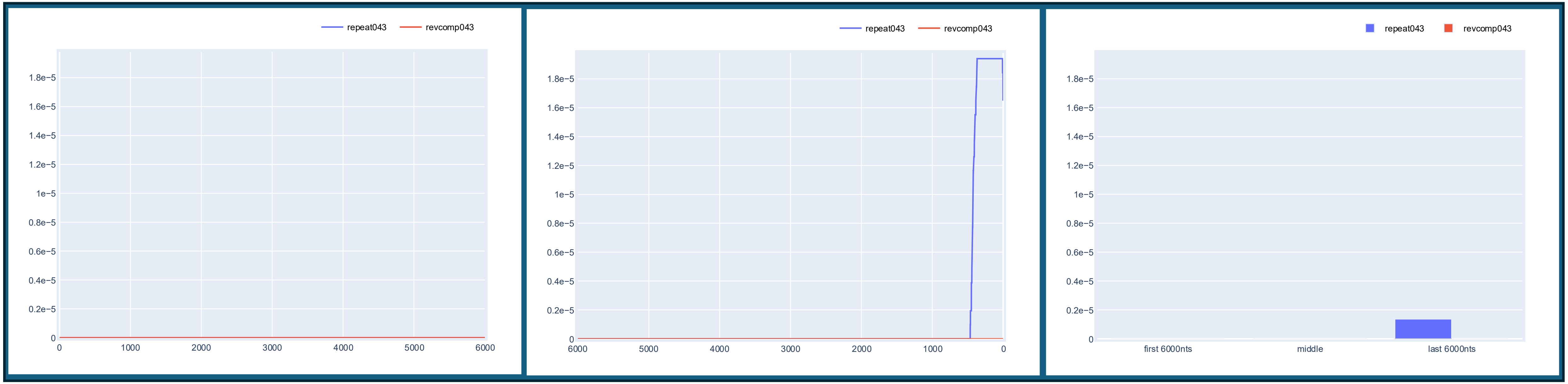

repeat pattern:
ACAGTTTCTCACCCCTGAAAAACACTGCATCAATAAAAATTTCA
AACTTATAAGTTCAGTAATCTCACAATAACACTAAAAATCAAACT
AGCA

reverse complement:
TGCTAGTTTGATTTTTAGTGTTATTGTGAGATTACTGAACTTATAAG
TTTGAAATTTTTATTGATGCAGTGTTTTTCAGGGGTGAGAAACTG
T

Repeat046 (occupancyMode; k51; K100; t1000; n6000 ERR10753927)

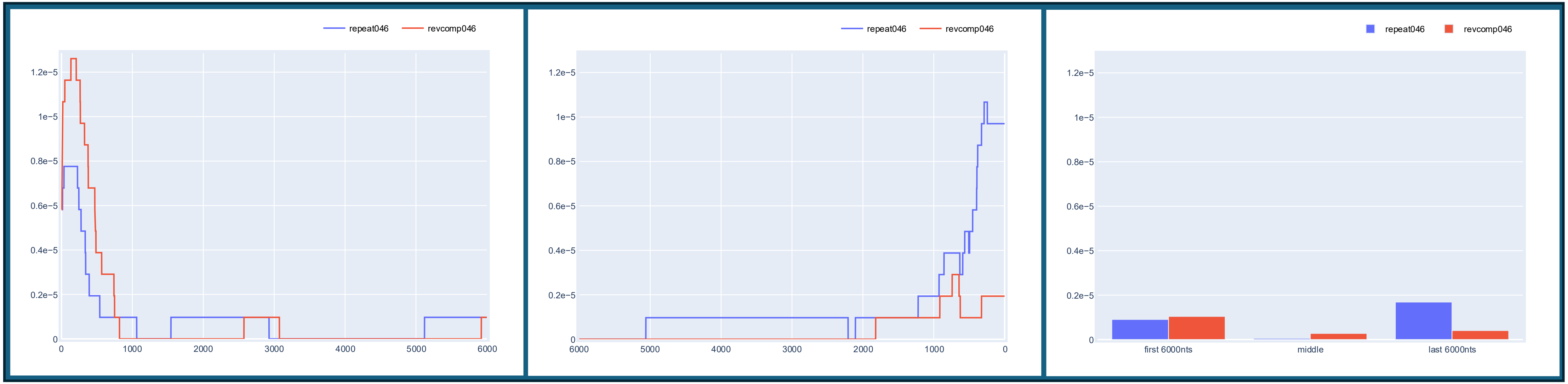

repeat pattern:
AAAAAAAAAAAAAAAAAAAAAAAAAAAAAAAAAAAAAAAAAAA
AAAAAAAA
reverse complement:
TTTTTTTTTTTTTTTTTTTTTTTTTTTTTTTTTTTTTTTTTTTTTTTTTTT

Repeat053 (occupancyMode; k51; K100; t1000; n6000 ERR10753927)

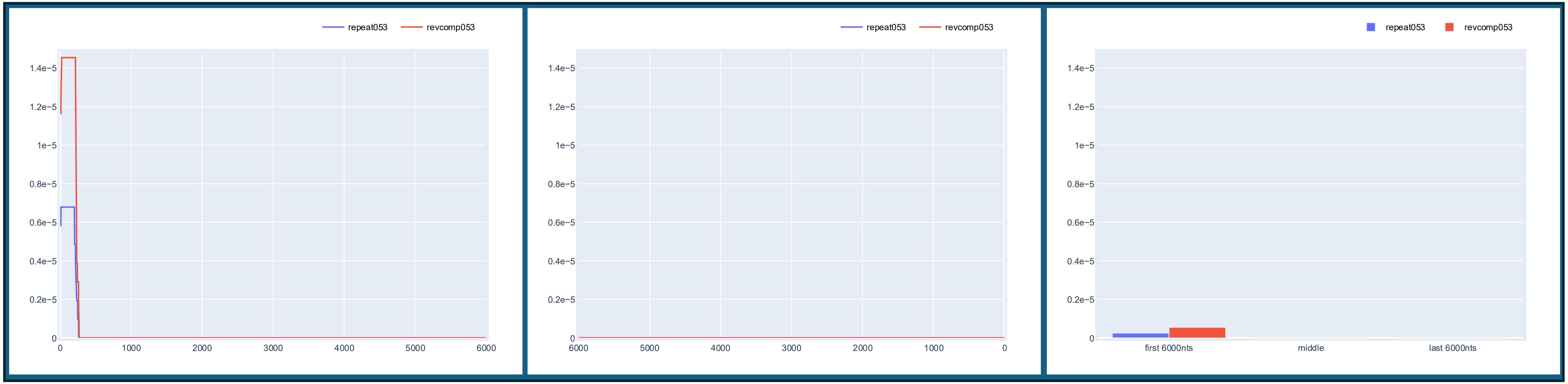

repeat pattern:
GCGTCAATAAAAAGGACAAACTTATAAGTTCAGCAATCTCTCAA
TAACACTAAAAATCAAACTAGCAACGGTTTCTCACCCCTGAAA
AACACT

reverse complement:
AGTGTTTTTCAGGGGTGAGAAACCGTTGCTAGTTTGATTTTTAGT
GTTATTGAGAGATTGCTGAACTTATAAGTTTGTCCTTTTTATTGAC
GC

Repeat056 (occupancyMode; k51; K100; t1000; n6000 ERR10753927)

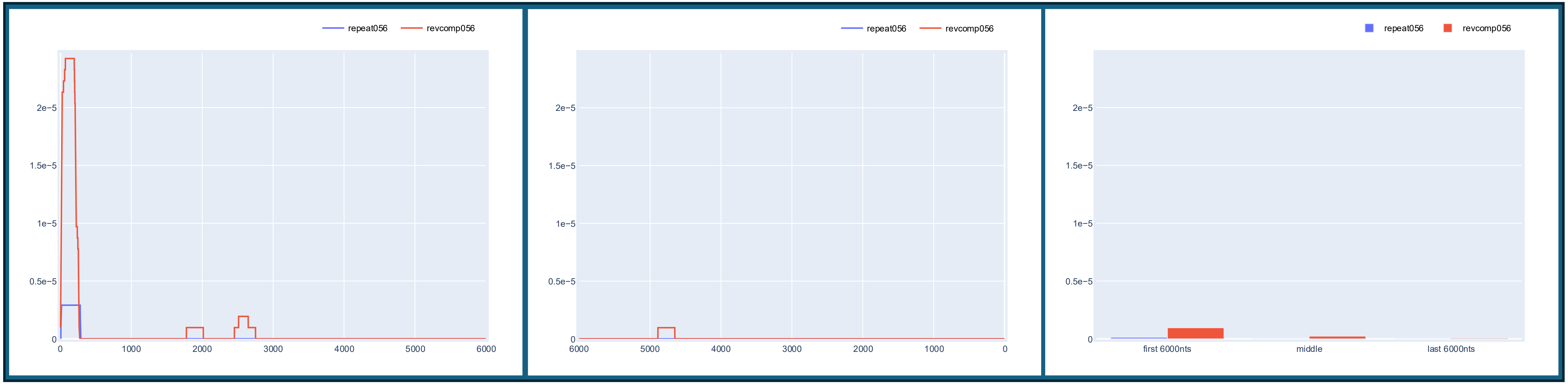

repeat pattern:
TAAGTTCAGTAATCTTACAGTAACACTAAAAATCAAACTAGCAA
CAGTTTCTCACCCCTGAAAAACACTGCGTCAATAAAAAGGAC
AAACTTA

reverse complement:
TAAGTTTGTCCTTTTTATTGACGCAGTGTTTTTCAGGGGTGAGAA
ACTGTTGCTAGTTTGATTTTTAGTGTTACTGTAAGATTACTGAACT
TA

Repeat058 (occupancyMode; k51; K100; t1000; n6000 ERR10753927)

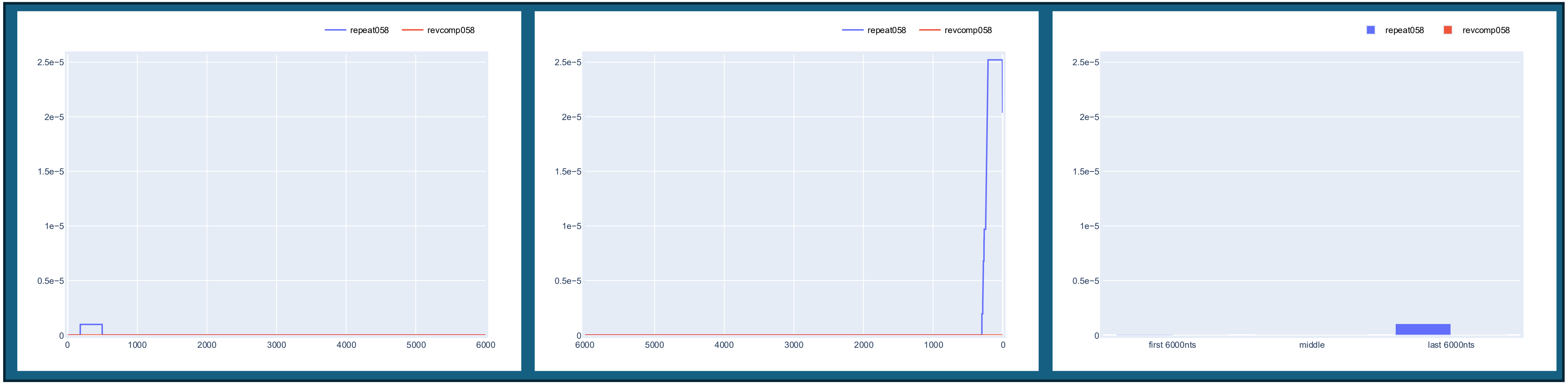

repeat pattern:
GCAACGGTTTCTCACCCCTGAAAAACACTGCGTCAATAAAGA
TTTCAAACTTATAAGTTCAGCAATCTCACAGTAAAACTAAAGGT
CAAACTA

reverse complement:
TAGTTTGACCTTTAGTTTTACTGTGAGATTGCTGAACTTATAAGTT
TGAAATCTTTATTGACGCAGTGTTTTTCAGGGGTGAGAAACCGT
TGC

Repeat069 (occupancyMode; k51; K100; t1000; n6000 ERR10753927)

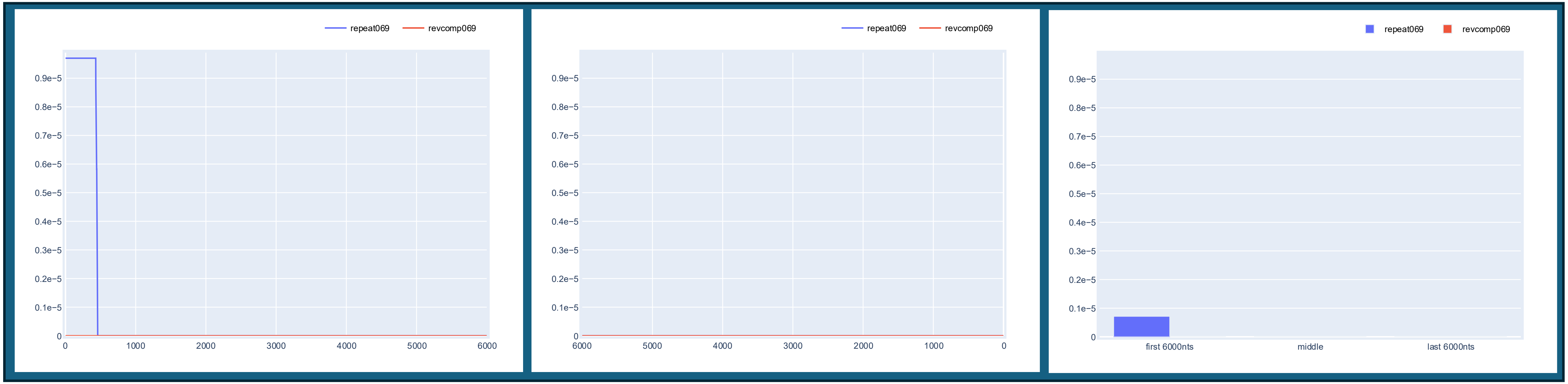

repeat pattern:
CTTTAGTGTTATTGAGAGATTGCTGAACTTATAAGTTTGAAATCTT
TATTGACGCAGTGTTTTTCAGGGGTGAGAAACCGTTGCTAGTTT
GAT
reverse complement:
ATCAAACTAGCAACGGTTTCTCACCCCTGAAAAACACTGCGT
CAATAAAGATTTCAAACTTATAAGTTCAGCAATCTCTCAATAACA
CTAAAG

Repeat072 (occupancyMode; k51; K100; t1000; n6000 ERR10753927)

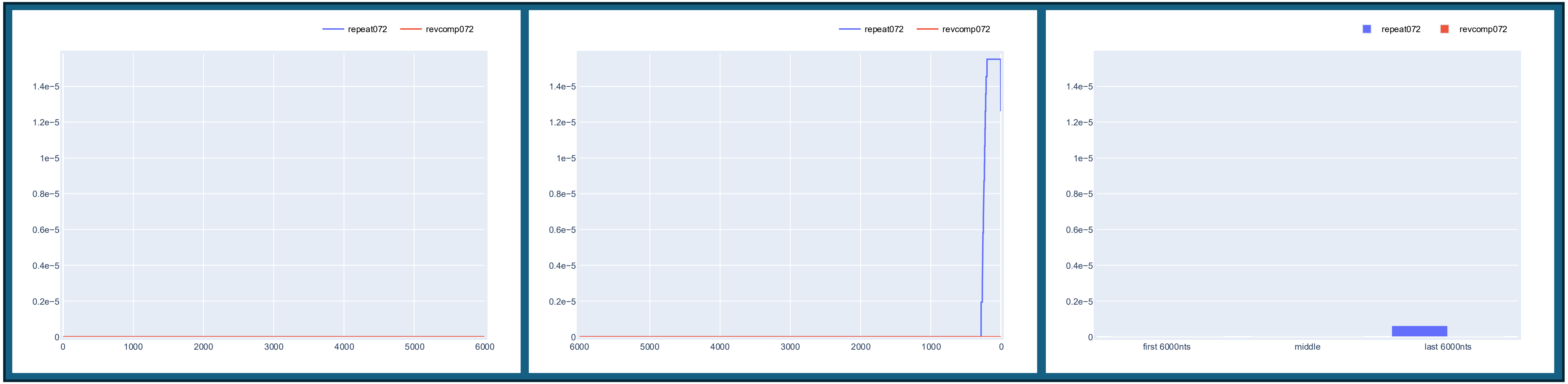

repeat pattern:
CGCAGTTTTTTTCAGAGGTGAGAAACCGTTGCTAGTTTGATCTT
TAGTGTTACTATGAGATTGCTGAACTTATAAGTTTGAAATCTTTATT
GA
reverse complement:
TCAATAAAGATTTCAAACTTATAAGTTCAGCAATCTCATAGTAAC
ACTAAAGATCAAACTAGCAACGGTTTCTCACCTCTGAAAAAAA
CTGCG

Repeat074 (occupancyMode; k51; K100; t1000; n6000 ERR10753927)

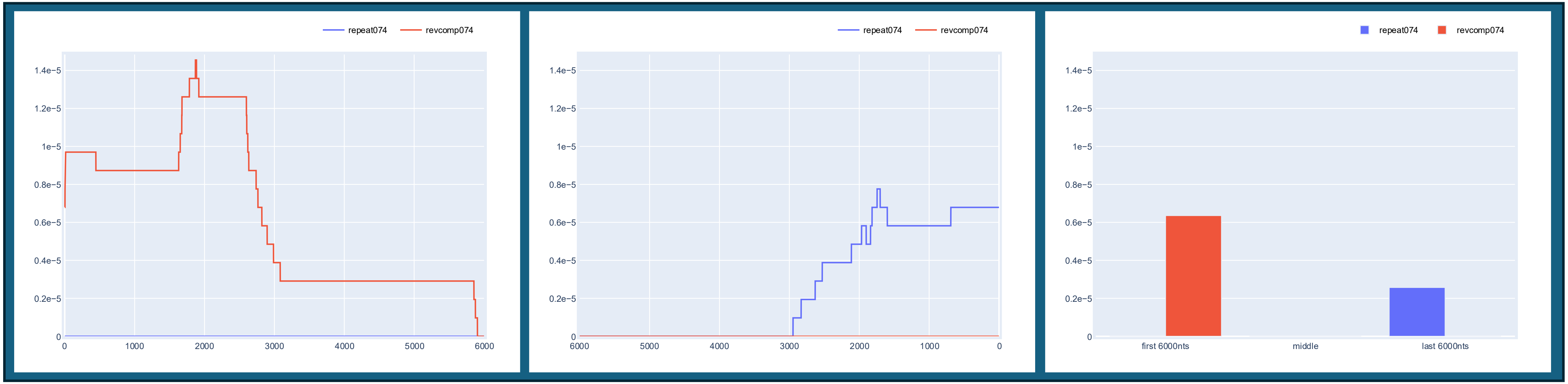

repeat pattern:
GGGTGGGTCGTTCCTGGGCCAAAGTCCGGGGGTGGGTGGTT
TTTTGGGCCGACGGGCCTGGTGG
reverse complement:
CCACCAGGCCCGTCGGCCCAAAAACCACCCACCCCCGG
ACTTTGGCCCAGGAACGACCCACCC

Repeat075 (occupancyMode; k51; K100; t1000; n6000 ERR10753927)

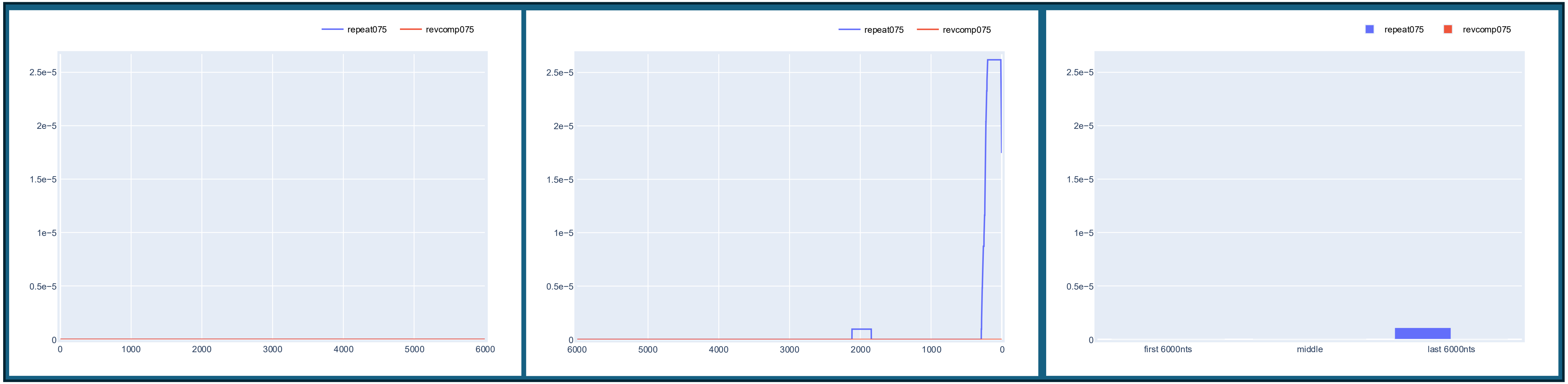

repeat pattern:
TAAGTTCAGCAATCTCATAGTACCACTAAAAACCAAACTAGCA
ACGGTTTCTCACCCCTGAAAAACACTGCGTCAATAAAAAGGA
CAAACTTA
reverse complement:
TAAGTTTGTCCTTTTTATTGACGCAGTGTTTTTCAGGGGTGAGAA
ACCGTTGCTAGTTTGGTTTTTAGTGGTACTATGAGATTGCTGAAC
TTA

Repeat080 (occupancyMode; k51; K100; t1000; n6000 ERR10753927)

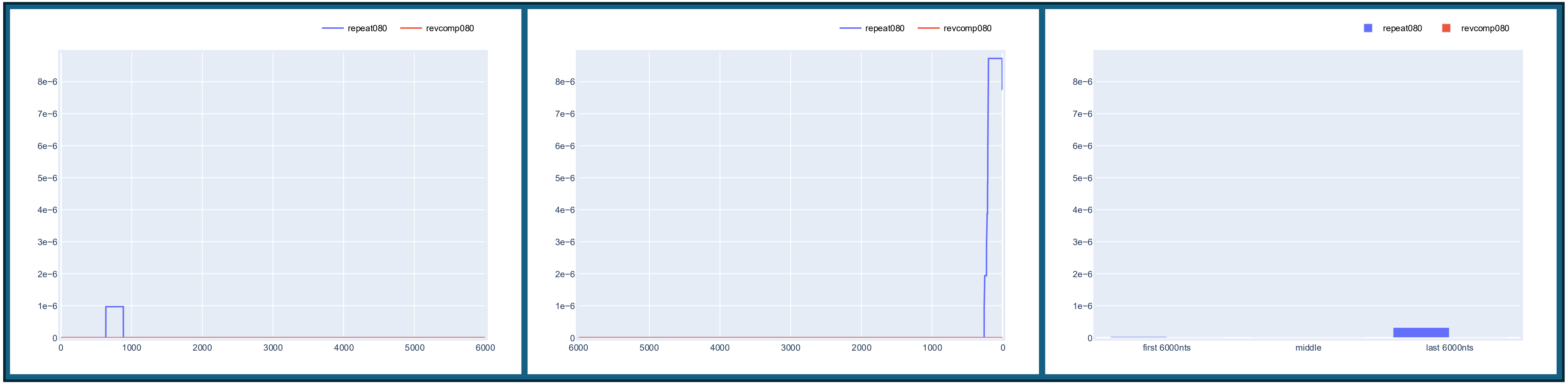

repeat pattern:
GCAGTTTTTTTCAGGGGCGAGAAACCGTTGCTAGTTTGATTTTT
AGTGTTACTGTGAGATTGCTGAACTTATAAGTTTGAAATCTTTATT
GAC

reverse complement:
GTCAATAAAGATTTCAAACTTATAAGTTCAGCAATCTCACAGTAA
CACTAAAAATCAAACTAGCAACGGTTTCTCGCCCCTGAAAAAAA
ACTGC

Repeat082 (occupancyMode; k51; K100; t1000; n6000 ERR10753927)

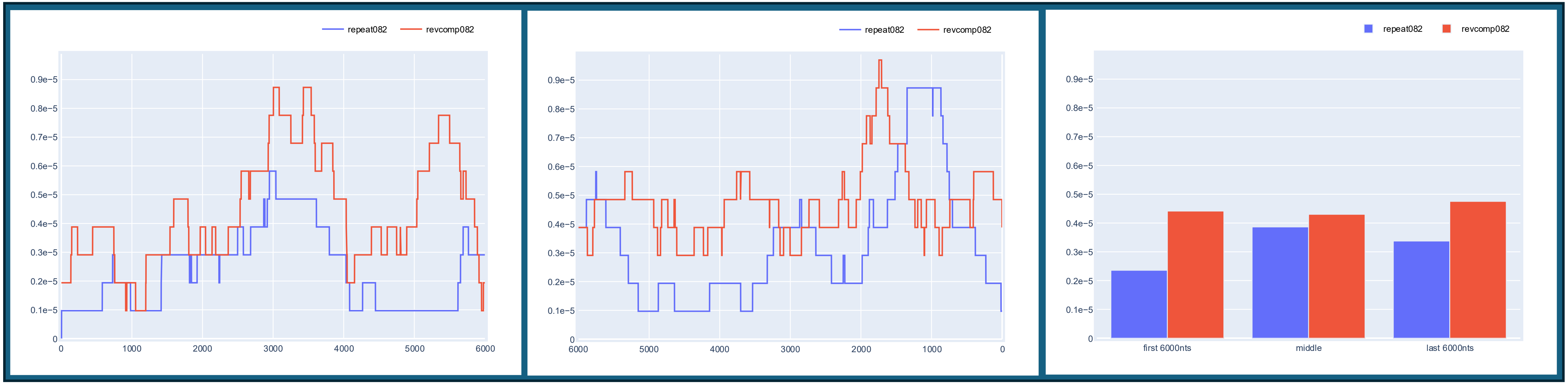

repeat pattern:
CAACAACTGACCATTTAAGGTGCAACAACTGACCATTTAAGGT
GCAACAACTGACCATTTAAGGTG
reverse complement:
CACCTTAAATGGTCAGTTGTTGCACCTTAAATGGTCAGTTGTTG
CACCTTAAATGGTCAGTTGTTG

Repeat090 (occupancyMode; k51; K100; t1000; n6000 ERR10753927)

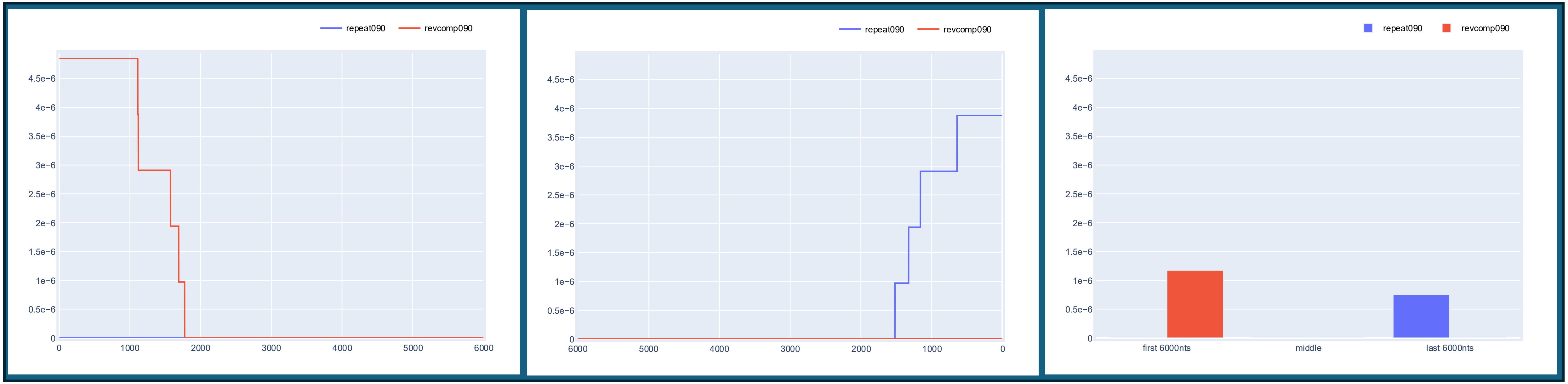

repeat pattern:
TGGGGGTGGGTCGTTCGGGGGGCAAGTTCCGGGGGTGGGT
CGTTTTTTGGGCCGACGGGCCTGG
reverse complement:
CCAGGCCCGTCGGCCCAAAAAACGACCCACCCCCGGAAC
TTGCCCCCCGAACGACCCACCCCCA

Repeat093 (occupancyMode; k51; K100; t1000; n6000 ERR10753927)

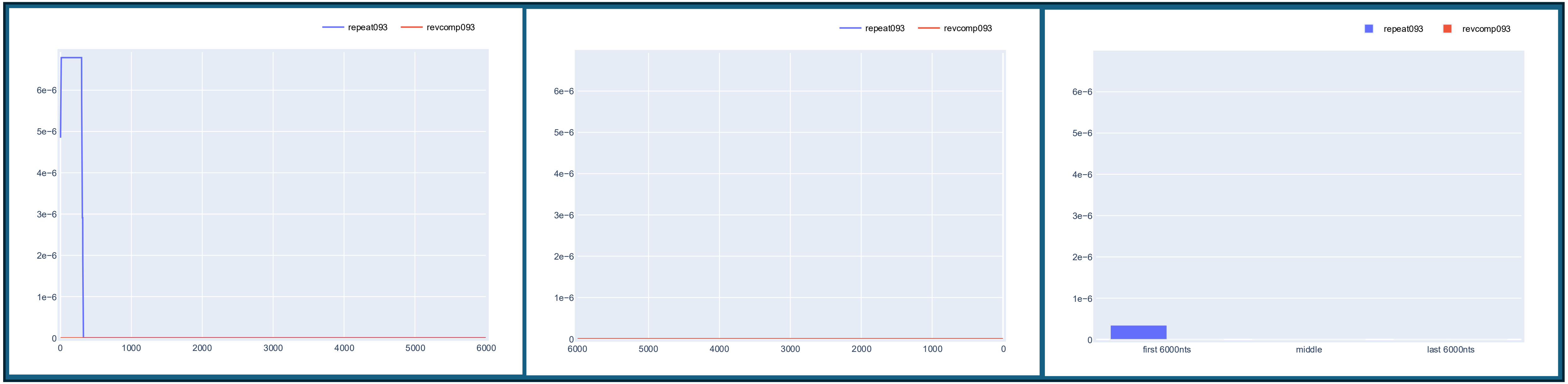

repeat pattern:
TAAGTTTGAAATCTTTATTGACGCAGTGTTTTTCAGGGGTGAGA
AACTGTTGCTAGTTTGATTTTTAGTGTTATTGAGAGATTACTGAAC
TTA

reverse complement:
TAAGTTCAGTAATCTCTCAATAACACTAAAAATCAAACTAGCAA
CAGTTTCTCACCCCTGAAAAACACTGCGTCAATAAAGATTTCA
AACTTA

Repeat098 (occupancyMode; k51; K100; t1000; n6000 ERR10753927)

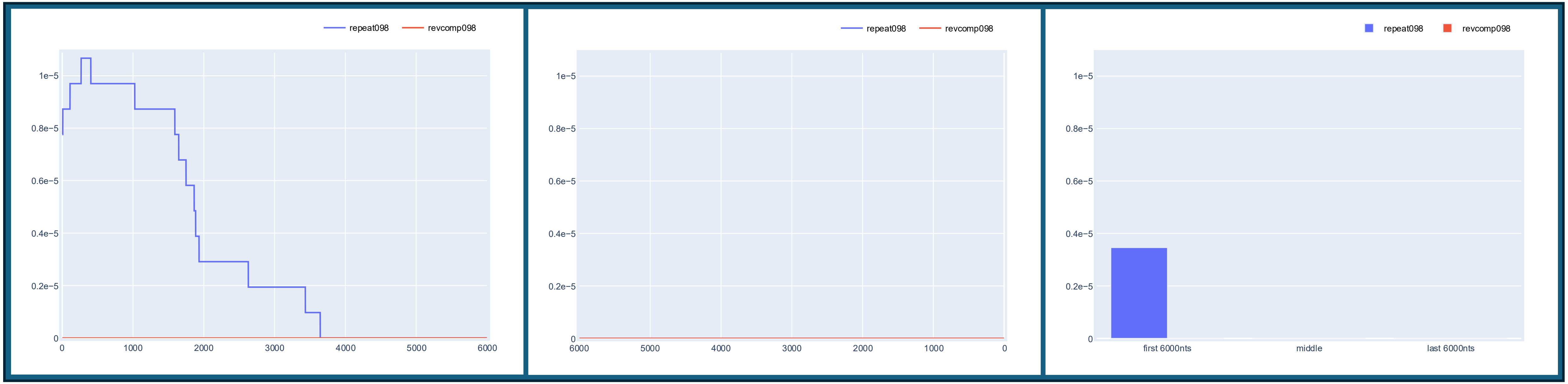

repeat pattern:
TCAGACCCGGAAATGACCCACCCCCGGAGTTTCGCCCGGA
TTCGACCCACCCCCACCAGGCCCATTTGACCCGAAAACGA
CCCACCCCCACCAAACCCA

reverse complement:
TGGGTTTGGTGGGGGTGGGTCGTTTTCGGGTCAAATGGGCCT
GGTGGGGGTGGGTCGAATCCGGGCGAAACTCCGGGGGTGG
GTCATTTCCGGGTCTGA

Repeat100 (occupancyMode; k51; K100; t1000; n6000 ERR10753927)

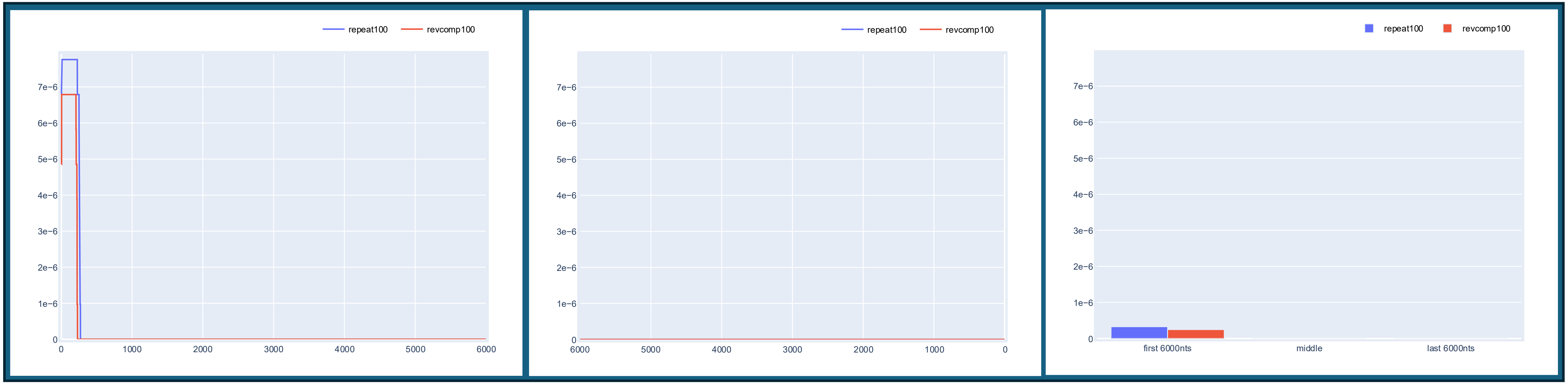

repeat pattern:
AACCGTTGCTAGTTTGACCTTTAGTGTTATTGAGAGATTGTTGAA
CTTATAAGTTTGTCCTTTTTATTGACGCAGTGTTTTTCAGGGGTG
AGA

reverse complement:
TCTCACCCCTGAAAAACACTGCGTCAATAAAAAGGACAAACTT
ATAAGTTCAACAATCTCTCAATAACACTAAAGGTCAAACTAGCA
ACGGTT

(A) TcTR1 and TcTR2 genes:

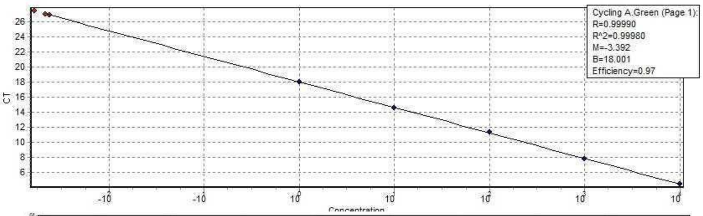

Standard curve: reaction efficiency
Template: Standars (plasmidDNA)
Primers: qTcTR1_F4 + qTcTr1_R1
Kit: LUNA kit, 60 °C

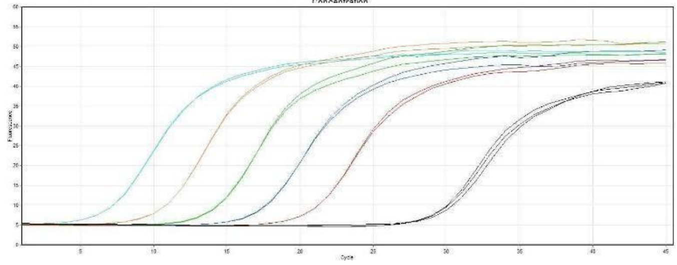

Standard curve: reaction efficiency
Template: Standars (plasmidDNA)
Primers: qTcTR1_F4 + qTcTr1_R1 **Kit:** LUNA kit, 60 °C

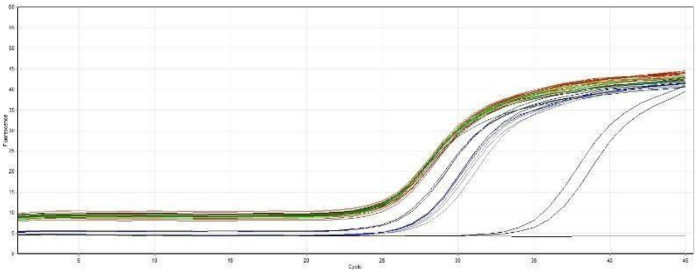

RT-qPCR of TcTR1
Template: cDNA of four *T.castaneum* stages (young larva, old larva, pupa and adult.
Primers: qTcTR1_F4 + qTcTr1_R1 **Kit:** LUNA kit, 60 °C

Primer qTcTR1_F4 = AGCATGGGTTTGTGAGCTT
Primer qTcTr1_R1 = TCTTTTGAGGGGAGAAAGGT

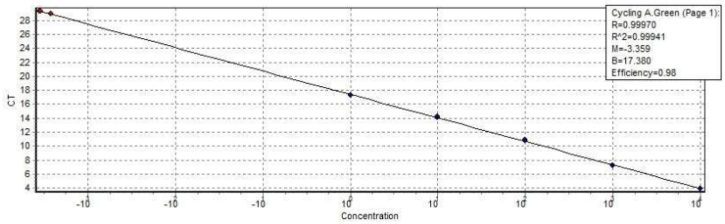

Standard curve: reaction efficiency
Template: Standars (plasmidDNA)
Primers: TcTR2_F2 + TcTr2_R3 **Kit:** LUNA kit, 60 °C

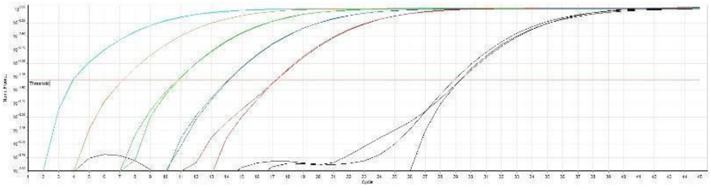

Standard curve: reaction efficiency
Template: Standars (plasmidDNA)
Primers: TcTR2_F2 + TcTr2_R3 **Kit:** LUNA kit, 60 °C

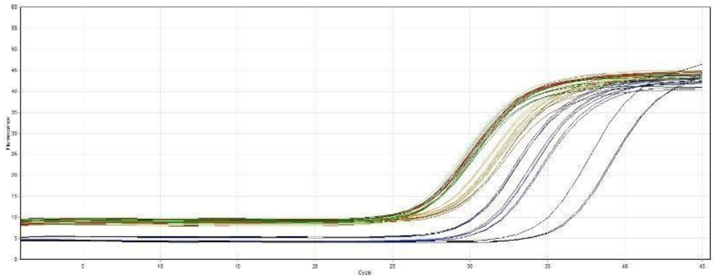

RT-qPCR of TcTR2
Template: cDNA of four *T.castaneum* stages (young larva, old larva, pupa and adult
Primers: qTcTR2_F2 + qTcTr2_R3
Kit: LUNA kit, 60 °C

Primer qTcTR2_F2 = GTTCGTTCAATGTGCAGTTC
Primer qTcTr2_R3 = GAGGGGAGAAATGTAGCAATTAC

(B) Reference gene ß-actin (ActB) (doi: 10.1093/ee/nvv010)

Primer TcACTb_fw**:** TCCATCATGAAGTGCGATGT
Primer TcACTb_rev: CCACATCTGTTGGAATGTCG
Amplicon length: 228 bp

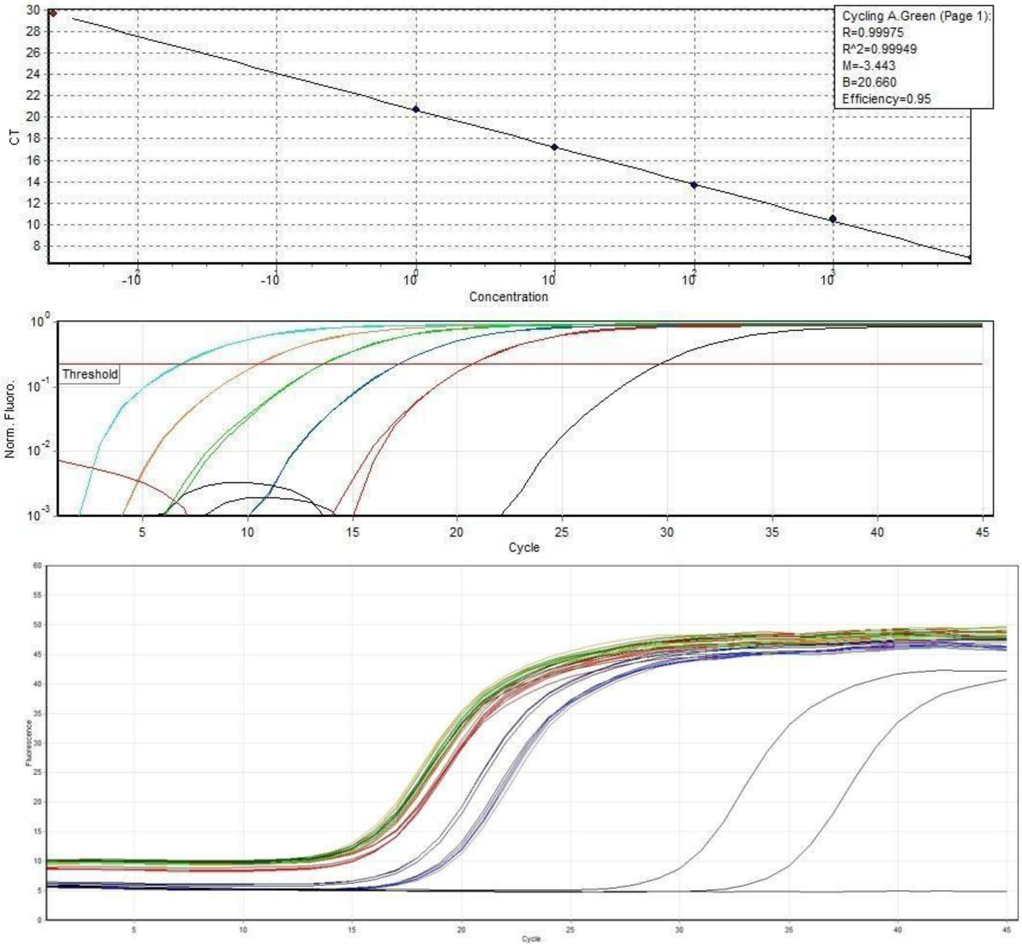

Supplementary Figure S5. qPCR efficiency validation for TcTR1, TcTR2 **(A)** and the reference gene β-actin (ActB) **(B)**. Amplification efficiency was evaluated using standard curves generated from serial dilutions of plasmid DNA templates. The resulting linear regression parameters and amplification plots confirm robust and comparable assay performance. RT-qPCR was performed on cDNA derived from four *Tribolium castaneum* developmental stages (young larva, old larva, pupa, and adult) using gene-specific primers. All reactions were conducted using the LUNA kit at 60 °C. Primer sequences are indicated.
